# Knowledge Driven Variable Selection (KDVS) – a new approach to enrichment analysis of gene signatures obtained from high–throughput data

**DOI:** 10.1186/1751-0473-8-2

**Published:** 2013-01-09

**Authors:** Grzegorz Zycinski, Annalisa Barla, Margherita Squillario, Tiziana Sanavia, Barbara Di Camillo, Alessandro Verri

**Affiliations:** 1 DIBRIS, University of Genoa, via Dodecaneso 35, I-16146 Genova, Italy; 2 Information Engineering Department, University of Padova, via Gradenigo 6A, I-35131 Padova, Italy

**Keywords:** Prior knowledge, High–throughput data, Gene signature, Enrichment analysis, Gene Ontology, Embedded Methods, ${\ell_{1}\ell_{2}}_{\text{FS}}$

## Abstract

**Background:**

High–throughput (HT) technologies provide huge amount of gene expression data that can be used to identify biomarkers useful in the clinical practice. The most frequently used approaches first select a set of genes (i.e. gene signature) able to characterize differences between two or more phenotypical conditions, and then provide a functional assessment of the selected genes with an *a posteriori* enrichment analysis, based on biological knowledge. However, this approach comes with some drawbacks. First, gene selection procedure often requires tunable parameters that affect the outcome, typically producing many false hits. Second, *a posteriori* enrichment analysis is based on mapping between biological concepts and gene expression measurements, which is hard to compute because of constant changes in biological knowledge and genome analysis. Third, such mapping is typically used in the assessment of the coverage of gene signature by biological concepts, that is either score–based or requires tunable parameters as well, limiting its power.

**Results:**

We present Knowledge Driven Variable Selection (KDVS), a framework that uses *a priori* biological knowledge in HT data analysis. The expression data matrix is transformed, according to prior knowledge, into smaller matrices, easier to analyze and to interpret from both computational and biological viewpoints. Therefore KDVS, unlike most approaches, does not exclude a priori any function or process potentially relevant for the biological question under investigation. Differently from the standard approach where gene selection and functional assessment are applied independently, KDVS embeds these two steps into a unified statistical framework, decreasing the variability derived from the threshold–dependent selection, the mapping to the biological concepts, and the signature coverage. We present three case studies to assess the usefulness of the method.

**Conclusions:**

We showed that KDVS not only enables the selection of known biological functionalities with accuracy, but also identification of new ones. An efficient implementation of KDVS was devised to obtain results in a fast and robust way. Computing time is drastically reduced by the effective use of distributed resources. Finally, integrated visualization techniques immediately increase the interpretability of results. Overall, KDVS approach can be considered as a viable alternative to enrichment–based approaches.

## Background

One of the challenges of modern molecular biology is to reconstruct the complex network of processes that govern all the activities of living organisms. HT technologies allow to measure the expression of thousands of genes simultaneously for each single biological sample [1,2], but these data are difficult to analyze because the number of samples is always lower with respect to the number of variables (e.g. genes, proteins). Therefore, traditional statistical methods are unsuitable to face this challenge. Nevertheless, these data are currently used to identify possible biomarkers, useful in the clinical practice: 1) to stratify a group of people affected by a disease into separated groups that are predicted to progress differently, 2) to subtype a disease through the identification of subgroups of people, that are characterized by a different molecular landscape, or by a different response to a specific drug. The probability of finding biomarkers is higher within the specific list of genes, known as gene signature [3,4], whose expression levels are able to separate two distinct phenotypic conditions (e.g. disease and healthy, tumor and metastasis) in comparison of two classes of biological samples. Such list can be identified in a typical binary classification setting [5].

Microarrays are a popular example of HT technology currently used to evaluate gene expression data that potentially contain meaningful gene signatures. The typical approach to analyze HT data is to first identify meaningful gene signatures and then to perform enrichment analysis [6,7].

Several methods can be chosen to identify gene signatures. The most commonly used filtering methods (e.g. t–test, ANOVA) [8,9] compare gene expression measurements to identify differentially expressed genes (DEG). The choice of the selection method is crucial because it affects the enrichment analysis. Specifically, if some relevant genes had not been included in the signature, it may not be possible to identify processes or functions that are connected with those genes, and that are relevant to the biological question addressed [10].

In the enrichment analysis, selected genes of the signature are compared with previously determined functional gene groups (that represent the current biological knowledge). To determine such groups, first it is necessary to assign genes to the biological concepts, that can be accomplished in various ways [11,12]. Then, the significant presence of genes in an individual group is assessed with some scoring schema or statistical test [6,13,14].

**Figure 1:**
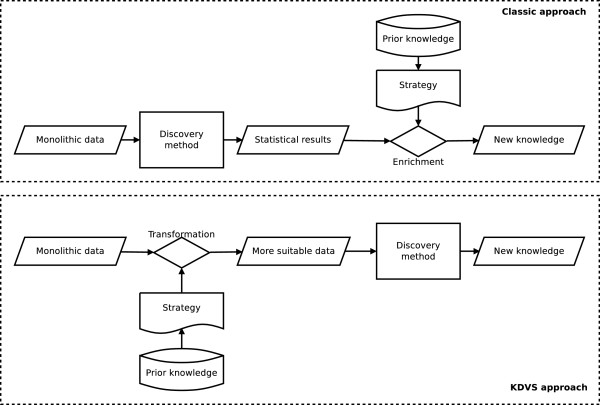
**The difference between classic approach and the KDVS approach.** Illustration of the difference between classic enrichment–based approach and the KDVS framework approach. In classic, monolithic gene expression data are mined for significant genes and prior biological knowledge is used a posteriori to verify the soundness of result. This approach is sensitive to the choice of mining technique, as well as the enrichment verification method. In KDVS, monolithic gene expression data are transformed according to prior knowledge (e.g. divided in smaller parts accordingly), and then mined for significant genes. This approach enables wider choice of mining techniques and provides biological insight before the core mining step.

Thus, in the classic enrichment analysis approach, the discovery method is not affected by the prior knowledge and it is performed independently with respect to the functional analysis. On the opposite, using prior knowledge before the selection step, the discovery method is applied according to the prior knowledge, thus giving a direct biological contextualization of the gene signature (Figure 1).

Several efforts in this direction have been recently presented in the literature. In [15], pathway information has been incorporated into logistic regression models. In [16-20] topological properties of Kyoto Encyclopedia of Genes and Genomes (KEGG) pathways have been used to constrain the learning process. The use of Gene Ontology (GO) as prior information has been explored in [21], where the authors propose a classification model based on functional groups of genes. A comparison of the performance obtained using different kind of knowledge has recently been presented in [22].

In this paper, we present KDVS (see Additional file 1), an implementation of the second case aforementioned, where the prior biological knowledge is used before the identification of the gene signature, the procedure itself is not altered, and the gene signature is defined according to the chosen source of prior knowledge. This framework can be considered as an alternative to most popular approaches of HT data analysis.

In contrast to other methods, KDVS accepts as input, besides the gene expression data, also specific annotations for HT platforms, and a representation of prior biological knowledge. As output, instead of gene signature and the results of the enrichment analysis, KDVS produces the list of significant biological concepts, as well as, for each of them, the list of significant genes that can be considered as individual signature associated to the concept.

The current implementation uses microarrays as source of gene expression data [23]. To analyze these data, among several supervised classification and variable selection methods [4,24], we choose ${\ell_{1}\ell_{2}}_{\text{FS}}$, an embedded regularization method for variable selection, proven to identify subsets of discriminative genes [25,26]. Gene Ontology (GO) is used as prior knowledge source [27].

KDVS is implemented in Python [28]. It was designed according to the principle of individual *applications* that share common functionality through the Application Programming Interface (API). Such modularized way allowed to dynamically adjust desired functionality piece by piece across a long time frame, which is crucial in the development of successful methodologies in statistical learning community. Also, it allowed to implement necessary solutions for standard bioinformatics–related problems, such as identifier mappings, in independent way. At the end, it allowed to seamlessly introduce the usage of parallel resources for time–consuming computations. Furthermore it is general enough to be applied to any other experiment that involves a two classes setting problem and it could be extended to a regression or multi-class setting.

The framework has been successfully applied to the analysis of microarray data in experiments regarding neurodegenerative diseases, namely Alzheimer’s and Parkinson’s diseases [29,30], as well as prostate cancer [31].

## Methods

### KDVS: Framework Overview

**Figure 2:**
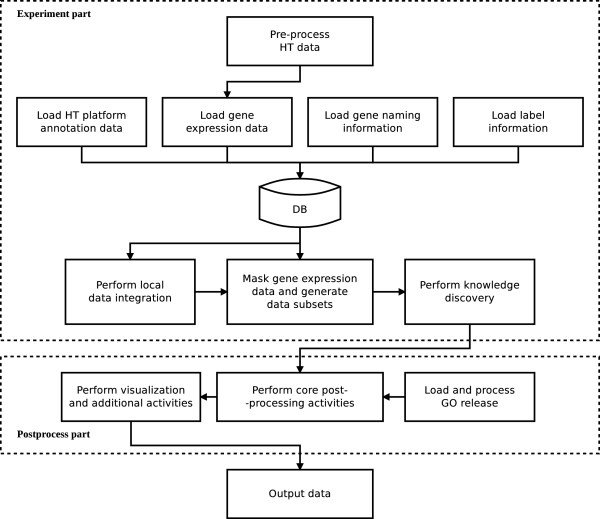
**General activity schema of KDVS.** Diagram of the general activity of KDVS.

The schema of the overall framework activity is presented in Figure 2. It is divided into *experiment part* and *postprocess part*. During the experiment part, all initial data processing takes place, all computational tasks are performed and the results are collected. The postprocess part works further on computational results to generate additional useful information and statistics. The experiment part is implemented in *experiment.py* and the postprocess part is implemented in *postprocess.py*.

#### Input data

The framework follows standard procedure for preparing microarray gene expression data [32]. The data are normalized using one of the standard methods available from BioConductor [33] set of packages (e.g. RMA, GCRMA, aroma). Its output is the Gene Expression Data Matrix (GEDM), in the form of Delimiter-Separated Values (DSV) file. The matrix has *P* rows and *N* + 1columns, where *P* is the number of genes/probe sets monitored over *N* biological samples, with the first column reporting the genes/probe sets IDs.

Moreover, the framework uses platform–specific annotations, that describe each measurement and all the related details (e.g. concerning the biological sequence used, the gene associated with it, its relation to other biological entities). In the case of microarray data, the framework utilizes chip–related annotations provided directly by the manufacturer or the research community in the form of a DSV file. KDVS uses platform annotations available from Gene Expression Omnibus (GEO) [34] for Affymetrix microarrays.

Since the framework heavily relies on prior biological knowledge, it utilizes a tailored representation of it. For example, for GO [27], the encoded Directed Acyclic Graph (DAG) structure is obtained, along with basic data regarding individual terms, such as the term identifier, the name and the description. In the current implementation, the framework uses the representation of GO graph, encoded in the RDF-XML file.

To perform supervised classification, it is necessary to associate each biological sample from the experiment with the phenotypical outcome, symbolized by specific *labels* that are often numerical [35]. For example, in the two class setting, samples can be associated with −1and 1 labels accordingly. The framework uses label information presented in the form of DSV file, where each sample is associated with the corresponding label.

#### Statistical analysis

The DSV data sets are loaded into individual tables (“raw” tables) of relational database. Specifically, a DSV data set containing *A* columns and *B* rows is loaded into the corresponding relational table that has *A* columns and contains *B* rows. In current implementation, GEDM matrix is loaded into “*GEDM*” table, the platform–related annotations is loaded into “*ANNO*” table, gene naming information is loaded into “*HGNC*” table, and the label information is loaded into “*LABELS*” table.

Querying raw tables directly may not be sufficient in some cases. To fulfill the main objective in effective way, some *derived* tables may be created: they contain recombined information from raw tables, and allow fast querying of raw data. For example, the Affymetrix annotations contain mapping *probeset*→*GO terms*. It is necessary, however, to obtain reverse mapping *GO term*→*probesets*, to associate each GO term with raw measurements. Therefore, the *term2probeset* derived table is created to allow fast querying of mapping *probeset*⇔*GO term* in both directions. However, platform–related annotations may not be up–to–date, regarding constant changes in annotations of genomes. This, for example, could affect gene naming (by presence of obsolete symbols), or coverage of genes by probesets (by rearrangement of gene sequences). Therefore another derived table, *probeset2gene*, is created to control the information from platform–specific annotations regarding gene naming, in accordance with the data obtained from the HUGO Gene Nomenclature Committee (HGNC) [36]. The process of construction of derived tables is also referred to as *local data integration*.

During the next step, *GEDM* and *term2probeset* tables are queried to generate specific expression data subsets that correspond to individual GO terms. The concept can be envisioned as *masking* the original monolithic GEDM data set and retrieving only the expression measurements that are associated with the specific GO term. The masking is repeated for each GO term in the *term2probeset* table that comes from the specified GO domain. Each data subset forms a matrix of *N* columns and *P*_*X*_ rows, where *P*_*X*_ is ||*P*. By construction, the data subsets overlap given the tree–structure of GO. Indeed each GO term may include several variables also belonging to other GO terms.

In the following step, a supervised classification is performed for each data subset. This step is also referred to as *knowledge discovery*, being the core part of recovering the functional characterization from gene expression data. In the current implementation, a variable selection technique ${\ell_{1}\ell_{2}}_{\text{FS}}$ is used [37-39]. The method returns the *classification error*, as well as the list of *selected variables* (in our case *probesets*), that are the most discriminant between two classes. The procedure depends on a *correlation* parameter *μ* that governs the amount of correlated variables selected in the final list. To evaluate an unbiased classification performance, ${\ell_{1}\ell_{2}}_{\text{FS}}$ performs a full *model selection* by using two nested *K*–fold cross–validation loops [39]. This also guarantees robustness, sensitivity and specificity in feature selection [40]. Data subset is further split into smaller parts, then classification is performed on those parts and finally these partial results are integrated into the final classification call. Based on that principle, the method counts the appearance of every single variable in each selected variable list obtained for each split, and it reports in addition the resulting frequency.

Where data subset is *sufficiently small*, that is, where the number of variables is roughly the same as number of samples, there is no need to perform full model selection. Thus, for those data subsets, a classification task is performed with Regularized Least Squares (RLS), and all variables are treated as selected or not, depending on the classification outcome.

The described statistical analysis focuses on the level of *probesets* instead of *genes*. This approach shows some considerable advantages. Typically, according to microarray design principles [41], the biological sequence of the gene can be monitored by more than one probeset. Therefore, to perform the analysis on the *gene* level, some method of aggregating the values of each probeset is required, e.g. by taking the average of the expression values. However, such procedures introduce certain additional bias into subsequent statistical analysis [35,42]. Since KDVS performs the analysis at the *probeset* level, there is no need for such procedures, and the mentioned additional bias is not introduced.

Given the machine learning procedure used for supervised classification, it may be desirable to utilize *a parallel computing environment* to speed up numerical computations [43]. In the current implementation, an ad–hoc environment was built over a local network of multi–core desktop machines, where the computational tasks were distributed to individual machines and executed in the background. The environment is controlled using the Python package PPlus [44].

The output of the experiment part consists of an *ensemble* of relational database and filesystem objects. The ensemble is used to store all the information used within the pipeline for further reference. This information includes: all the input data, the results from local data integration, all the generated data subsets and the output from knowledge discovery. The output from knowledge discovery becomes the direct input for the next part.

#### Postprocess phase

This part is roughly composed of the *core post–processing activities* and the *visualization* tasks.

During *core activities*, the results obtained from the *knowledge discovery* step are reviewed and an *error estimate* is obtained. Here, if the classification error is *below* the chosen threshold, the GO term associated with a specific data subset is considered *significant*. Next, for each significant term, the absolute frequency of each of its selected variables is checked: if the frequency is *above* the specified threshold, the variable is considered *properly selected*. The frequency is checked across variables selected in the outer *K*–fold cross validation loop during the *model selection* phase (see [39] for details). Next, several useful statistics are collected, such as the standard properties of classification error (i.e. the mean, the standard deviation, the median, the histograms of selected and non–selected variables across all data subsets). Basic plots of classification errors are generated as well.

Furthermore, each variable (here defined as probeset) is *annotated* back with biologically meaningful annotations, such as the gene symbol related to the probeset, and the identifiers of the corresponding Entrez Gene [45] and Genbank [46] database records. The annotations come primarily from the *ANNO* table, and are verified against *HGNC* table with gene naming information [36], if applicable.

The output of the *core activities* of the postprocess step is the list of *significant GO terms* that pass the supervised classification step, and for each significant GO term, the list of *properly selected* probesets. The classification results allow focusing on specific biological functionality represented by the GO terms, and the frequencies allow focusing only on those genes determined as playing important role in a biological experiment. Even if the output obtained during the *core activities* in the postprocess part is meaningful, its further biological analysis may be difficult, due to its low interpretability for researchers outside machine learning and statistics communities. The introduction of the visualization step is crucial to represent the results in a more visually clear way and it is useful to perform further biologically–oriented investigations.

To this aim, significant GO terms are visualized on the minimal subgraph built over the complete DAG of specific GO domain [47,48]. The subgraph is generated with the *GOstats* BioConductor [33] package and visualized with R interface to *GraphViz* graph plotting library [49].

In order to further increase the interpretability of the results, a *semantic clustering* was introduced. Referring to the DAG, for each GO term the Information Content [50] is calculated by comparing the number of occurring annotations in the graph subsumed by that term to the total number of annotations belonging to the GO graph subsumed by the root node. This metric is based on frequency of the term occurring in annotations by taking advantage of the structural information organized in GO; thus a rarely used term in the GO graph contains a greater amount of information. Starting from this metric, it is possible to define a semantic similarity measure in order to assess the degree of relatedness between two GO terms. There are different similarity measures based on this kind of metric [51]. Here, Resnik semantic similarity was used since it associates to a pair of terms the information content of their common ancestor, thus preserving the level of specificity of the relationships between the terms. For the implementation of both the information content and the Resnik measure the standalone *FastSemSim* Python application [52] was used. FastSemSim requires as input data the description of the DAG and the GO annotations. This application allows other different semantic similarity measures, thus the user can also decide to use a different metric. However, since the aim is to group the selected GO terms into homogeneous clusters, a normalized measure is required. In this case, Resnik measure was normalized to the maximum observed value.

Given the list of significant GO terms defined in the postprocess step, a semantic similarity matrix was build from the Resnik pairwise similarities provided by FastSemSim. The similarity matrix was then used as input to perform a hierarchical clustering. In order to select the clusters, a default semantic threshold equal to 0.8 was used in the implementation, which can be changed by the user. Finally, the resulting clusters of terms were plotted on the minimal subgraph with different colors using *GOstats* and *GraphViz*.

#### Output data

The main interpretable output of KDVS consists of the lists of *significant GO terms*, and for each significant term, the list of *properly selected* variables (determined during *Postprocess phase*).

Significant GO terms are obtained both with ${\ell_{1}\ell_{2}}_{\text{FS}}$ and RLS techniques. For ${\ell_{1}\ell_{2}}_{\text{FS}}$, a list of significant terms is generated for each used *μ* value [39], and for RLS a single list is generated. Since ${\ell_{1}\ell_{2}}_{\text{FS}}$ procedure involves full model selection as well as variable selection, some meaningful details are collected here for each significant GO term as well. To obtain a consistent output, each list of GO terms obtained with ${\ell_{1}\ell_{2}}_{\text{FS}}$ for given *μ* value, is merged with single list of GO terms obtained with RLS, thus making *unified term lists*.

The specific details noted for significant GO terms obtained with ${\ell_{1}\ell_{2}}_{\text{FS}}$ include: the index of proper ${\ell_{1}\ell_{2}}_{\text{FS}}$*μ*value, mean and standard deviation of test error, mean and standard deviation of training error, median of test error, total number of variables in corresponding data subset, and number of properly selected variables. Mean test error is later re–used for unified term list as classification error estimate. ${\ell_{1}\ell_{2}}_{\text{FS}}$ should guarantee that the classification error estimate does not change much for different values of *μ*. An abbreviated example output list of significant GO terms obtained with ${\ell_{1}\ell_{2}}_{\text{FS}}$ is presented below:

Each unified term list consists of significant GO terms obtained with ${\ell_{1}\ell_{2}}_{\text{FS}}$ for single *μ*value, and significant GO terms obtained with RLS. The following details are collected for each term: its full name, total number of variables in corresponding data subset, number of properly selected variables, classification error estimate used for significance call, numbers of: true positives, true negatives, false positives, and false negatives, collected across outer *K*–fold cross–validation loop, as well as the corresponding Matthews Correlation Coefficient (MCC). An abbreviated example of unified term list is presented below:

For significant GO terms, obtained either with ${\ell_{1}\ell_{2}}_{\text{FS}}$ or RLS, a list of *properly selected* variables is generated. The following details are noted for each properly selected variable: its corresponding probeset symbol, gene symbol (verified on the *ANNO* and *HGNC* tables), its corresponding Entrez Gene ID and Genbank record ID, as well as the frequency checked across outer cross–validation loop (if obtained with ${\ell_{1}\ell_{2}}_{\text{FS}}$). For ${\ell_{1}\ell_{2}}_{\text{FS}}$, the variables list is generated for the GO term only if this term is considered significant for that particular ${\ell_{1}\ell_{2}}_{\text{FS}}$*μ* value. For RLS, the details are similar, only frequency is omitted, since variable selection is not performed. Note that some corresponding external IDs may not be present. An abbreviated example output list of properly selected variables is presented below:

Regarding variables, two histograms are also constructed. In the first case, for each variable present in original data set, it is counted how many times that variable was selected in data subsets correspoding to significant GO terms. In the second case, also for each variable in original data set, it is counted how many times that variable was *not* selected in data subsets correspoding to significant GO terms. In both cases, the following details are recorded for each variable: its corresponding probeset ID, gene symbol, and the respective count for that variable. Also noted are: the total number of entries in the histogram, the number of significant GO terms, and the number of GO terms in the GO domain considered. For ${\ell_{1}\ell_{2}}_{\text{FS}}$–related data subsets, we produce one histogram for each *μ*value. For RLS–related data subsets, we produce single histogram for selected variables, since for corresponding significant GO terms *all* variables are properly selected. An abbreviated sample histogram of variables selected at least once is presented below:

Besides interpretable output, KDVS also collects technical output, such as binary files, additional diagnostic files and logs.

### KDVS: Framework Architecture

**Figure 3:**
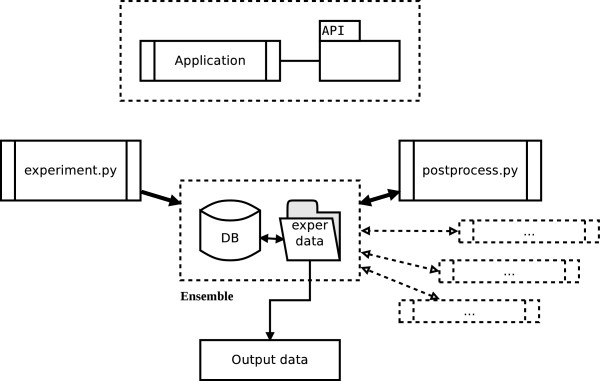
**Schema of the architecture of KDVS.** KDVS consists of applications that work on common ensemble of data. Each application uses the KDVS API component.

The architecture of the framework is presented in Figure 3. All the involved applications work on a single *ensemble* of information, that consists of a relational database and filesystem objects. The ensemble is first obtained by *experiment.py*, and then modified by subsequent applications.

The default relational database engine, available for Python *SQLite*[53], is used due to its simplicity of usage, low memory footprint and low maintenance needs. Each database can contain many tables and is stored as single file. Currently, a single database is created for each experiment.

#### Applications in KDVS

Each application in KDVS is composed as a sequence of *actions* that are executed within specified *execution environment*. Actions are Python procedures. In this case, actions can use *shared variables*, maintained by the environment, to transfer their state to other actions. The most basic execution environment provides uniform logging and error handling; more complicated environments can provide access to distributed resources, interface to external applications etc.

Applications use KDVS *API* to perform their tasks: some functionalities shared by all applications, as well as some specific functionality considered stable are placed into separate Python package kdvs.

#### experiment.py

**Figure 4:**
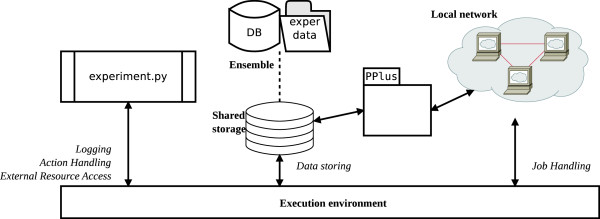
**Activity schema of experiment.py application.** The experiment.py application creates ensemble of data, performs data integration and transformation, manages distributed computational environment and performs knowledge discovery procedure.

The *experiment.py* application generates the ensemble, performs local data integration and knowledge discovery. The general activity schema is presented in Figure 4.

We used L1L2Py [54], a Python implementation of ${\ell_{1}\ell_{2}}_{\text{FS}}$, that contains also an implementation of RLS (note that we use RLS with regularization parameter equal to 0). Since ${\ell_{1}\ell_{2}}_{\text{FS}}$ is quite time–consuming because it performs a full model selection, *experiment.py* manages a simple environment for distributed computations. The environment consists of a group of multi–core machines, connected over a local network, that accept and execute computational tasks. Each *split*, performed by ${\ell_{1}\ell_{2}}_{\text{FS}}$, is a single task that is distributed and executed. The environment utilizes *shared storage*, a dedicated machine that exposes storage device available over the local network. The environment, as a whole, is managed by the PPlus Python library [44], that encapsulates the access to shared storage as simple file operations, and controls distribution and execution of computational tasks. PPlus was built on top of Parallel Python (PP) [55], that provides a simple solution for managing local group of machines that execute Python code in tasks.

To speed up the process of building the ensemble, only the data that are crucial for execution of computational tasks are put on shared storage and accessed from there. The relational database is managed uniquely on the machine that started *experiment.py* application. Later, the data from the shared storage are copied back to the same location as database file and the ensemble is considered complete.

#### postprocess.py

**Figure 5:**
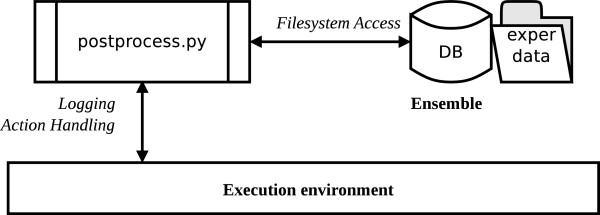
**Activity schema of postprocess.py application.** The postprocess.py application performs supplementary activities on ensemble of data (e.g. assembling final results, collecting useful statistics).

The *postprocess.py* application works on the completed ensemble to identify the functional output of the framework and to generate useful miscellaneous data for the experiment. The general activity schema is presented in Figure 5.

Since this application does not have high time requirements, it uses very simple execution environment.

#### Additional scripts

Some independent R and Python scripts are used with the KDVS framework, to perform functionalities that are either not easily accomplished with Python alone or are considered the standard in its domain.

To perform normalization of Affymetrix microarray data, standalone R script is used (see Additional file 2 for an example). It uses standard BioConductor [33] normalization methods (i.e. RMA, GCRMA, aroma) and is tailored separately for each biological experiment.

A standalone R script is used (see Additional file 3 for an example) to perform visualization of the minimal subgraph of GO graph. This script utilizes GOstat and RGraphViz packages and is tailored separately for the specific list of GO terms obtained in the experiment.

To perform semantic clustering, the standalone fastsemsim Python package script (see Additional file 4 for an example) is used. It is tailored separately for the specific list of GO terms obtained in the experiment.

### Enrichment analysis

For comparison purposes, KDVS results were compared to classic enrichment–based approaches [25,26]. To this aim, we used WebGestalt, an online gene set analysis toolkit [56] taking as input a list of relevant genes/probesets and performing an enrichment analysis in KEGG and GO, identifying the most relevant pathways and ontologies in the signatures.

### Benchmark list

In order to evaluate the biological soundness of the results, we identified two benchmark lists, namely genes and corresponding GO terms, that refer to the current state of knowledge for the disease of interest. Together, they provide a reference way to evaluate the biological consistency of our results.

The benchmark gene list is the union of three lists available from public repositories, namely KEGG PATHWAY [57], KEGG DISEASE [57], and the Gene Prospector tool [58]. The first two sources refer to experimentally validated results, while the last one contains also the results of up–to–date literature mining of genetic association studies. We chose those prior knowledge sources because they are explicitly associated with the investigated diseases.

The benchmark list of GO terms was obtained by identifying associated GO terms, according to Gene Ontology Annotation (GOA) data [27], for each gene contained in the benchmark gene list. We chose this method because, to our knowledge, there are no established sources of GO terms that correspond to specific diseases.

The results produced by enrichment–based approach and KDVS are based on different methodologies, even if the same discovery method – ${\ell_{1}\ell_{2}}_{\text{FS}}$ – is used. Therefore, instead of comparing them directly, we chose to make pairwise comparisons between the list of genes and list of discriminant GO terms, produced by each approach, and the respective benchmark lists.

In case of gene lists, the benchmark coverage is calculated for all benchmark genes. In case of GO term lists, however, it is necessary to acknowledge the fact that KDVS produces the results only for single GO domain at the time. Therefore, during the calculation of the benchmark coverage of GO terms, we recognize two cases. For enrichment–based approach, we consider all benchmark GO terms, while for KDVS we consider only the benchmark terms from that specific GO domain used in the experimental setting.

All benchmark lists used in this work are available as Additional file 5. The detailed description of the workflow followed to make the benchmark data is provided as Additional file 6.

## Results and discussion

### Prostate cancer study

We applied KDVS to analyze the GSE6919 [59,60] microarray dataset available in GEO. The dataset contains gene expression data from metastatic, primary tumor and control prostate tissue samples processed on a platform measuring the expression of about 10.000 genes. After the normalization and the quality control steps, performed using the *aroma* package and the *arrayQualityMetrics* R packages [33], we analyzed 25 metastatic, 63 primary tumor and 80 normal tissue samples. We addressed two classification problems: one concerning the characterization of metastasis and primary tumor and one discriminating between primary tumor and normal tissue.

In preliminary results [31], only the first classification problem was addressed using Molecular Function (MF) as source of GO domain knowledge.

Successively each classification problem was addressed twice, considering as prior knowledge MF and Biological Process (BP) GO domains. Those GO terms whose classification error was below 30% were considered discriminant for both GO domains. The genes associated with these terms were merged and redundant genes were discarded. For each classification problem, the framework identified two lists of GO terms (one for MF and one for BP) and two gene lists, respectively. In addition, we addressed the same classification problems using classic enrichment–based approach (see Table 1). In this case, in the binary classification problem comparing primary tumor and metastasis, the 4–fold cross–validation error was 1%. In the other problem, the 5–fold cross–validation error was 27%.

**Table 1:** Results of the KDVS for Prostate cancer study

**Kind**	**Enrichment**		**KDVS**			
**Lists**	**PTvsM**	**PTvsN**	**PTvsM**	**PTvsM**	**PTvsN**	**PTvsN**
			**MF**	**BP**	**MF**	**BP**
**Discr. GO Terms**	60	120	1115	2242	320	689
**Discr. Genes**	59	389	3619	4457	3118	3271
**Comm. GO Terms**	27	61	375	1000	158	378
**Comm. Genes**	7	51	418	504	334	371
**Bench. Cov. GO Terms**	1%	2%	46%	41%	19%	16%
**Bench. Cov. Genes**	1%	6%	49%	59%	39%	44%

In the first two rows, the table shows the discriminant gene and GO term lists identified by enrichment–based approach and KDVS for each classification problem solved: Primary Tumor (PT) versus Metastasis (M) and Primary Tumor vs Normal (N). For KDVS, in addition, each classification task was addressed two times, based on the GO domain utilized, Molecular Function (MF) or Biological Process (BP). Discr. stands for discriminant.

In the next two rows, the table shows the gene and GO term lists obtained as an intersection between respective discriminant lists and benchmark lists, built for prostate cancer. Comm. means common.

In the last two rows, the table shows the coverage of benchmark gene and GO term lists with respective discriminant lists. For KDVS, the coverage was calculated only for benchmark MF and BP terms, respectively, according to GO domain utilized in classification task. Benchmark gene list consists of 851 elements. Benchmark GO term list consists of 2437 BP terms and 824 MF terms, 3593 terms in total. Bench. cov. means benchmark coverage.

In order to evaluate the biological soundness of the results, we identified two benchmark lists, namely genes and the corresponding GO terms, that refer to the current state of knowledge for prostate cancer (see Additional files 5 and 6). The benchmark gene list consists of 851 elements. The benchmark GO term list consists of 2437 BP terms and 824 MF terms, 3593 terms in total.

The comparison of our results with the benchmark lists shows the better performance of KDVS, as shown in Table 1. High percentage of genes and GO terms identified by KDVS were already known to be associated with the disease. Therefore, these results increase the likelihood that the remaining identified knowledge could be related with the biological issue under investigation.

### Parkinson’s disease study I

In another case study, first presented in [30], we analyzed the microarray dataset GSE20295 [61,62], also available from GEO. It is composed of Parkinson’s disease (PD) samples in advanced stage of development and control sample tissues, 40 and 53 cases respectively, measured on a platform that includes about 33.000 genes. The MF domain of GO was chosen as source of prior knowledge to decompose GEDM into data subsets, associated with their relative GO terms. The terms whose classification error was below 30%, were considered as discriminant between the two classes. As reported in [30], these terms were found to be correlated with the disease under study, and their representation as subgraph provided a way to better visualize the results. In addition, we addressed the same classification problem using the classic enrichment–based approach (see Table 2). In this case, the 8–fold cross–validation error was 21%.

**Table 2:** Results of the KDVS for Parkinson disease study I & II

**Kind**	**Enrichment**		**KDVS**	
**Lists**	**PDvsN I**	**PDvsN II**	**PDvsN I**	**PDvsN II**
**Discr. GO terms**	77	65	364	150
**Discr. Genes**	77	66	5705	4286
**Comm. GO Terms**	31	31	113	54
**Comm. Genes**	9	3	274	196
**Benchmark Cov. GO Terms**	2%	1%	25%	12%
**Benchmark Cov. Genes**	2%	1%	62%	44%

In the first two rows, the table shows the discriminant gene and GO term lists identified by enrichment–based approach and KDVS for Parkinson study I and II, respectively. Both studies were performed on two different microarray datasets of Parkinson (PD) and Normal (N) tissue samples. Discr. means discriminant.

In the next two rows, the table shows the gene and GO term lists obtained as an intersection between respective discriminant lists and benchmark lists, built for Parkinson’s disease. Comm. means common.

In the last two rows, the table shows the coverage of benchmark gene and GO term lists with respective discriminant lists. For KDVS, the coverage was calculated only for benchmark MF terms, according to the nature of classification task. Benchmark gene list consists of 444 elements. Benchmark GO term list consists of 2121 terms in total, including 446 MF terms. Bench. cov. means benchmark coverage.

Both the list of GO terms and the corresponding list of genes were successfully compared with the benchmark lists for PD, that contain 2121 GO terms and 444 genes respectively. The benchmark lists were obtained following the same procedure as described in Prostate cancer study (see Additional files 5 and 6).

As shown in Table 2, significant number of genes and GO terms identified by KDVS were already known to be associated with PD. In addition, the KDVS results show greater coverage of benchmark data with respect to the enrichment–based approach.

### Parkinson’s disease study II

The last case study regards the analysis of GSE6613, a microarray dataset [63], available from GEO. The dataset is composed of 33 early stage Parkinson samples and 22 controls, measured on a platform that includes about 33.000 genes. The MF domain of GO was chosen as source of prior knowledge to decompose GEDM into data subsets, associated with their relative GO terms. Also in this study, the terms whose classification error was below 30%, were considered as discriminant between the two classes. In addition, we addressed the same classification problem using the classic enrichment–based approach (see Table 2). In this case, the 8–fold cross–validation error was 36%.

For comparison, we used the same benchmark lists as for Parkinson’s disease study I.

Table 2 shows that both approaches were able to extract sound biological knowledge, nonetheless the results obtained from KDVS underline a higher coverage of the benchmark knowledge, with respect to those obtained by the classic enrichment–based approach.

### KDVS conceptual framework

KDVS pipeline presented here is one instance of a conceptual framework that integrates prior knowledge with standard machine-learning-based selection procedures.

In [64] a similar method was proposed, evaluating the predictive performance of functional categories based on Leave-One-Out Regularized Least Squares (LOO-RLS). The authors also aimed at assessing the statistical significance of each predictor exploiting the closed form solution for LOO-RLS and a multiple random validation strategy. Despite presenting a very similar concept, KDVS adds the feature selection phase combined with the prediction assessment. This is particularly useful especially when the number of variables is very high with respect to the number of samples, which is a very common situation for a large number of GO terms.

In particular, we considered ${\ell_{1}\ell_{2}}_{\text{FS}}$ as selection method. Nevertheless, KDVS is, by design, flexible to incorporate the selection method of choice: ${\ell_{1}\ell_{2}}_{\text{FS}}$ could be replaced by any other embedded or wrapper variable selection method based on classification [65,66] or filter methods combined with a prediction step [67].

Finally, it is worth remarking that KDVS aims at using the existing knowledge as additional information to structure the data matrix before the analysis step, while enrichment methods such as GSEA, Random sets, GLAPA and others [13,68] incorporate the domain knowledge *a posteriori*.

## Conclusions

The KDVS framework has been developed to demonstrate the advantages of using prior biological knowledge before identifying the list of GO terms and genes statistically discriminant between two classes in the analysis of high-throughput data. Therefore it is presented as an alternative method to the most frequently used approaches that use prior knowledge a posteriori, once the gene signature has been identified. Its modular structure allows quick adaptation to different HT data types, prior knowledge sources, and different classification and variable selection approaches. The usage of distributed resources overcomes demanding computational requirements that are common in HT data analysis (microarray, NGS, etc.). Furthermore the conceptual integration of the visualization approach in the framework activities determines greater interpretability of the results, especially for life science researchers.

In the future, we plan to make tighter integration with existing semantic clustering approach, to introduce different sources of prior biological knowledge, to accept different HT data, and to introduce wide range of classification and variable selection techniques.

## Abbreviations

HT: high-throughput; KDVS: Knowledge Driven Variable Selection; DEG: differentially expressed genes; GO: Gene Ontology; API: Application Programming Interface; GEDM: Gene Expression Data Matrix; DSV: Delimiter Separated Values; GEO: Gene Expression Omnibus; DAG: Directed Acyclic Graph; RDF: Resource Description Framework; XML: Extensible Markup Language; HGNC: HUGO Gene Nomenclature Committee; RLS: Regularized Least Squares; MCC: Matthews Correlation Coefficient; FS: Feature Selection; PP: Parallel Python; GOA: Gene Ontology Annotations; MF: Molecular Function; BP: Biological Process; PD: Parkinson’s disease; LOO-RLS: Leave-One-Out-Regularized Least Squares; NGS: next–generation sequencing.

## Competing interests

The authors declare that they have no competing interests.

## Authors’ contributions

GZ conceived, designed, and implemented the KDVS framework. AB designed the study, developed statistical methodology, and supervised KDVS development. MS provided biological insight. TS and BDC contributed to the ideas of prior knowledge usage and of semantic clustering. AV developed statistical methodology and supervised the study. All authors have contributed to the manuscript. All authors read and approved the final manuscript.

## Supplementary Material

Additional file 1 **Source code of KDVS.** Format: ZIP. It contains the Python source code, the documentation, and the internal data files. Click here for file

Additional file 2 **Example script performing microarray data normalization and quality check.** Format: R. The proper input directory and chip type must be provided. The script uses aroma folder structure to identify input data. Click here for file

Additional file 3 **Example script producing visualization artifacts.** Format: R. The proper input directory and GO domain must be specified. The script scans KDVS ensemble for postprocessing data and visualizes minimal subgraphs for the list of selected GO terms. Click here for file

Additional file 4 **Example script performing semantic clustering.** Format: Python. The script accepts single DSV output files generated by KDVS, containing list of significant GO terms, and performs semantic clustering with *fastsemsim* Python library. Proper data files used by *fastsemsim*, as well as clustering details, must be provided. The clustering is performed with default settings, using *scipy.cluster.hierarchy.fcluster*. For more details see *fastsemsim* documentation. Click here for file

Additional file 5 **Benchmark lists for Prostate Cancer and PD.** Format: XLS. The spreadsheet contains the benchmark lists of genes and GO terms, considered trustable enough to use in comparisons between enrichment–based results and KDVS results. See Additional file 6 for more information. Click here for file

Additional file 6 Detailed description of the construction of benchmark data. Click here for file
