# Supplementary material for: Knowledge Driven Variable Selection (KDVS) – a new approach to enrichment analysis of gene signatures obtained from high–throughput data
Source: Source Code Biol Med. 2013 Jan 9;8:2. doi: 10.1186/1751-0473-8-2 (PMC3605163; doi:10.1186/1751-0473-8-2)
Supplement: Additional file 1 — Source code of KDVS. Format: ZIP. It contains the Python source code, the documentation, and the internal data files. [file 1751-0473-8-2-S1.zip › KDVS/doc/_build/html/doc-api/GO_GEDM.html]

kdvs.core.GO.GEDM — KDVS 0.0.1-alpha documentation


### Navigation

- index
- modules |
- modules |
- next |
- previous |
- KDVS 0.0.1-alpha documentation »
- KDVS API »

# kdvs.core.GO.GEDM¶

Provides functionality for manipulating gene expression data matrix (GEDM).

kdvs.core.GO.GEDM.get\_GEDM\_probesets(*source\_db*)¶
:   Get all probesets associated with GEDM (usually row IDs) in load order.

    |  |  |
    | --- | --- |
    | Parameters : | **source\_db** : db\_provider  tablespace that contains table ‘GEDM’ |
    | Returns : | **probesets** : iterable  probesets associated with GEDM in load order |

kdvs.core.GO.GEDM.get\_GEDM\_rows(*source\_db*, *probesets=None*, *samples=None*)¶
:   Get rows of expression values associated with given GEDM probesets, filter
    columns according to GEDM samples if needed.

    |  |  |
    | --- | --- |
    | Parameters : | **source\_db** : db\_provider  tablespace that contains table ‘GEDM’  **probesets** : iterable/None  probesets for which expression values are to be retrieved, or None if to retrieve values for all probesets at once (affects rows of the table)  **samples** : iterable/None  samples for which expression values are to be retrieved, or None if to retrieve values for all samples at once (affect columns of the table) |
    | Returns : | **probeset2row** : dict  dictionary that maps probesets to rows of related expression values |

    Notes

    To get whole GEDM matrix, use probesets=None and samples=None.

kdvs.core.GO.GEDM.get\_GEDM\_samples(*source\_db*, *id\_col=False*)¶
:   Get all samples associated with GEDM (usually column IDs) in load order.

    |  |  |
    | --- | --- |
    | Parameters : | **source\_db** : db\_provider  tablespace that contains table ‘GEDM’  **id\_col** : bool  if False, skip the first column ID (it may not be associated with any sample) |
    | Returns : | **samples** : iterable  samples associated with GEDM in load order |

### Quick search


Enter search terms or a module, class or function name.

### Navigation

- index
- modules |
- modules |
- next |
- previous |
- KDVS 0.0.1-alpha documentation »
- KDVS API »

© Copyright 2010-2012, Grzegorz Zycinski, Salvatore Masecchia, Annalisa Barla.
Created using Sphinx 1.1.2.
