# Supplementary material for: Knowledge Driven Variable Selection (KDVS) – a new approach to enrichment analysis of gene signatures obtained from high–throughput data
Source: Source Code Biol Med. 2013 Jan 9;8:2. doi: 10.1186/1751-0473-8-2 (PMC3605163; doi:10.1186/1751-0473-8-2)
Supplement: Additional file 1 — Source code of KDVS. Format: ZIP. It contains the Python source code, the documentation, and the internal data files. [file 1751-0473-8-2-S1.zip › KDVS/doc/_build/html/doc-api/GO_GOTermTree.html]

kdvs.core.GO.GOTermTree — KDVS 0.0.1-alpha documentation


### Navigation

- index
- modules |
- modules |
- next |
- previous |
- KDVS 0.0.1-alpha documentation »
- KDVS API »

# kdvs.core.GO.GOTermTree¶

Provides functionality for parsing RDF-XML release of Gene Ontology data into
GO term tree.

kdvs.core.GO.GOTermTree.parse\_go\_rdf\_xml(*xmlfilename*, *termurl='http://www.geneontology.org/go#'*)¶
:   Parse RDF-XML file provided by GO that contains descriptions of GO terms,
    including terms hierarchy.

    |  |  |
    | --- | --- |
    | Parameters : | **xmlfilename** : string  path to RDF-XML file containing descriptions of GO terms  **termurl** : string  prefix of unique URL assigned to each GO term |
    | Returns : | **terms** : dict  dictionary containing mapping of parent GO terms to children GO terms  **synonyms** : dict  dictionary containing synonymous terms present for some GO terms |

### Quick search


Enter search terms or a module, class or function name.

### Navigation

- index
- modules |
- modules |
- next |
- previous |
- KDVS 0.0.1-alpha documentation »
- KDVS API »

© Copyright 2010-2012, Grzegorz Zycinski, Salvatore Masecchia, Annalisa Barla.
Created using Sphinx 1.1.2.
