# Supplementary material for: Knowledge Driven Variable Selection (KDVS) – a new approach to enrichment analysis of gene signatures obtained from high–throughput data
Source: Source Code Biol Med. 2013 Jan 9;8:2. doi: 10.1186/1751-0473-8-2 (PMC3605163; doi:10.1186/1751-0473-8-2)
Supplement: Additional file 1 — Source code of KDVS. Format: ZIP. It contains the Python source code, the documentation, and the internal data files. [file 1751-0473-8-2-S1.zip › KDVS/doc/_build/html/doc-api/GO_GOTermTreeManip.html]

kdvs.core.GO.GOTermTreeManip — KDVS 0.0.1-alpha documentation


### Navigation

- index
- modules |
- modules |
- next |
- previous |
- KDVS 0.0.1-alpha documentation »
- KDVS API »

# kdvs.core.GO.GOTermTreeManip¶

Provides functionality for manipulating Gene Ontology term tree.

## See Also¶

GOTermTree

kdvs.core.GO.GOTermTreeManip.collect\_immediate\_children(*termtree*, *synonyms*, *node*)¶
:   Given GO term, collect all of its immediate children.

    Immediate children of term X are GO terms related directly with “is\_a” or “part\_of”
    relations with X.

    |  |  |
    | --- | --- |
    | Parameters : | **termtree** : dict  dictionary containing GO term hierarchy in the form of mapping parent GO terms to children GO terms  **synonyms** : dict  dictionary containing existing synonymous terms present for some of GO terms  **node** : string  current GO term being processed |
    | Returns : | **children** : iterable  immediate children of requested node |

kdvs.core.GO.GOTermTreeManip.collect\_subtree\_terms(*termtree*, *synonyms*, *root\_node*)¶
:   Collect all nodes from rooted GO term subtree into a set.

    |  |  |
    | --- | --- |
    | Parameters : | **termtree** : dict  dictionary containing GO term hierarchy in the form of mapping parent GO terms to children GO terms  **synonyms** : dict  dictionary containing existing synonymous terms present for some of GO terms  **root\_node** : string  root of the GO term subtree to process |
    | Returns : | **terms** : set  set of GO terms from rooted subtree |

kdvs.core.GO.GOTermTreeManip.collect\_subtree\_terms\_with\_depth(*termtree*, *synonyms*, *root\_node*)¶
:   Collect all nodes from rooted GO term subtree into a set, as well as mapping
    of numerical depth level to list of nodes at that level.

    |  |  |
    | --- | --- |
    | Parameters : | **termtree** : dict  dictionary containing GO term hierarchy in the form of mapping parent GO terms to children GO terms  **synonyms** : dict  dictionary containing existing synonymous terms present for some of GO terms  **root\_node** : string  root of the GO term subtree to process |
    | Returns : | **terms** : set  set of GO terms from rooted subtree  **termdepth** : dict  dictionary containing mapping of depth level to corresponding nodes |

kdvs.core.GO.GOTermTreeManip.collect\_term\_parents(*termtree*, *synonyms*, *node*)¶
:   Given GO term, collect all of its parents.

    Parents of term X are GO terms related directly with “is\_a” or “part\_of”
    relations with X.

    |  |  |
    | --- | --- |
    | Parameters : | **termtree** : dict  dictionary containing GO term hierarchy in the form of mapping parent GO terms to children GO terms  **synonyms** : dict  dictionary containing existing synonymous terms present for some of GO terms  **node** : string  current GO term being processed |
    | Returns : | **parents** : iterable  parents of requested node |

kdvs.core.GO.GOTermTreeManip.subtree(*termtree*, *synonyms*, *node*, *depth*, *function*, *\*args*, *\*\*kwargs*)¶
:   Given tree of GO terms, execute given callback function on the requested node
    on the given level of the GO term subtree. Proceed with recursive calls for
    every child of the requested node.

    Callback function accepts the requested node, list of children of the requested
    node and current level depth. Any additional arguments are passed to callback
    function through variable argument list. For example:

    ```
    def dump_to_outfile(node, children, depth, *args, **kwargs):
        # dump tree hierarchy to the output file
        file = args[0]
        print >> file, node.rjust(len(node) + depth), '-->', children
    ```

    |  |  |
    | --- | --- |
    | Parameters : | **termtree** : dict  dictionary containing GO term hierarchy in the form of mapping parent GO terms to children GO terms  **synonyms** : dict  dictionary containing existing synonymous terms present for some of GO terms  **node** : string  current GO term being processed  **depth** : integer  current level of the GO term subtree  **function** : callable  callback function object  **args** : iterable  any additional positional arguments passed to callback function  **kwargs** : dict  any additional keyworded arguments passed to callback function |

### Table Of Contents

- kdvs.core.GO.GOTermTreeManip
  - See Also

### Quick search


Enter search terms or a module, class or function name.

### Navigation

- index
- modules |
- modules |
- next |
- previous |
- KDVS 0.0.1-alpha documentation »
- KDVS API »

© Copyright 2010-2012, Grzegorz Zycinski, Salvatore Masecchia, Annalisa Barla.
Created using Sphinx 1.1.2.
