# Supplementary material for: Knowledge Driven Variable Selection (KDVS) – a new approach to enrichment analysis of gene signatures obtained from high–throughput data
Source: Source Code Biol Med. 2013 Jan 9;8:2. doi: 10.1186/1751-0473-8-2 (PMC3605163; doi:10.1186/1751-0473-8-2)
Supplement: Additional file 1 — Source code of KDVS. Format: ZIP. It contains the Python source code, the documentation, and the internal data files. [file 1751-0473-8-2-S1.zip › KDVS/doc/_build/html/doc-api/GO_HGNC.html]

kdvs.core.GO.HGNC — KDVS 0.0.1-alpha documentation


### Navigation

- index
- modules |
- modules |
- next |
- previous |
- KDVS 0.0.1-alpha documentation »
- KDVS API »

# kdvs.core.GO.HGNC¶

Provides functionality for manipulating gene naming-related data (http://www.genenames.org/).

kdvs.core.GO.HGNC.create\_probeset2gene(*kdvsdata*, *expdb\_id*, *hgnc\_table\_name*, *gpl\_table\_name*, *dst\_table\_schema*, *gpl\_metadata*, *hgnc\_metadata*)¶
:   Create derived table probeset2gene based on imported DSV data.

    |  |  |
    | --- | --- |
    | Parameters : | **kdvsdata** : KDVSDB  instance of KDVS DB manager  **expdb\_id** : db\_provider  tablespace that already contains tables ‘ANNO’ and ‘HGNC’; probeset2gene table will be created there  **hgnc\_table\_name** : string  proper name of ‘HGNC’ table  **gpl\_table\_name** : string  proper name of ‘ANNO’ table  **dst\_table\_schema** : iterable  detailed schema of probeset2gene table  **gpl\_metadata** : KDVSMetadata  KDVS metadata used to parse specific portions of data from ‘ANNO’ table  **hgnc\_metadata** : KDVSMetadata  KDVS metadata used to parse specific portions of data from ‘HGNC’ table |

    See also

    db, annotation\_metadata

kdvs.core.GO.HGNC.get\_geneid(*source\_db*, *probeset*)¶
:   Get gene naming-related data for requested GEDM probeset.

    |  |  |
    | --- | --- |
    | Parameters : | **source\_db** : db\_provider  tablespace that contains table ‘probeset2gene’  **probeset** : string  name of requested probeset |
    | Returns : | **geneid** : tuple  tuple (gene symbol, Entrez Gene ID, GenBank accession number) for requested GEDM probeset |

kdvs.core.GO.HGNC.get\_probeset2geneid(*source\_db*)¶
:   Get mapping of GEDM probesets to gene-naming related data.

    |  |  |
    | --- | --- |
    | Parameters : | **source\_db** : db\_provider  tablespace that contains table ‘probeset2gene’ |
    | Returns : | **probeset2geneid** : dict  dictionary that maps GEDM probesets to corresponding gene naming-related data in the following way:  ``` probeset -> [[gene_symbols] [entrez_ids] genbank_acc] ```  where gene\_symbols and entrez\_ids are in load order |

    See also

    get\_geneid()

### Quick search


Enter search terms or a module, class or function name.

### Navigation

- index
- modules |
- modules |
- next |
- previous |
- KDVS 0.0.1-alpha documentation »
- KDVS API »

© Copyright 2010-2012, Grzegorz Zycinski, Salvatore Masecchia, Annalisa Barla.
Created using Sphinx 1.1.2.
