# Supplementary material for: Knowledge Driven Variable Selection (KDVS) – a new approach to enrichment analysis of gene signatures obtained from high–throughput data
Source: Source Code Biol Med. 2013 Jan 9;8:2. doi: 10.1186/1751-0473-8-2 (PMC3605163; doi:10.1186/1751-0473-8-2)
Supplement: Additional file 1 — Source code of KDVS. Format: ZIP. It contains the Python source code, the documentation, and the internal data files. [file 1751-0473-8-2-S1.zip › KDVS/doc/_build/html/doc-api/GO_annotation.html]

kdvs.core.GO.annotation — KDVS 0.0.1-alpha documentation


### Navigation

- index
- modules |
- modules |
- next |
- previous |
- KDVS 0.0.1-alpha documentation »
- KDVS API »

# kdvs.core.GO.annotation¶

Provides functionality for manipulating vendor-specific annotation data for
microarrays.

kdvs.core.GO.annotation.create\_term2probeset(*kdvsdata*, *expdb\_id*, *gpl\_table\_name*, *dst\_table\_schema*, *gpl\_metadata*)¶
:   Create derived table term2probeset based on imported DSV data.

    |  |  |
    | --- | --- |
    | Parameters : | **kdvsdata** : KDVSDB  instance of KDVS DB manager  **expdb\_id** : db\_provider  tablespace that already contains table ‘ANNO’; term2probeset table will be created there  **gpl\_table\_name** : string  proper name of ‘ANNO’ table  **dst\_table\_schema** : iterable  detailed schema of term2probeset table  **gpl\_metadata** : KDVSMetadata  KDVS metadata used to parse specific portions of data from ‘ANNO’ table |

    See also

    db, annotation\_metadata, go\_metadata

kdvs.core.GO.annotation.get\_ns\_terms(*source\_db*, *go\_namespace*)¶
:   Get all GO terms available for requested GO namespace.

    |  |  |
    | --- | --- |
    | Parameters : | **source\_db** : db\_provider  tablespace that contains table ‘term2probeset’  **go\_namespace** : string  requested GO namespace |
    | Returns : | **terms** : iterable  GO terms available for requested GO namespace |

kdvs.core.GO.annotation.get\_probesets(*source\_db*)¶
:   Get all probesets available through term2probeset mapping.

    |  |  |
    | --- | --- |
    | Parameters : | **source\_db** : db\_provider  tablespace that contains table ‘term2probeset’ |
    | Returns : | **probesets** : iterable  probesets available through term2probeset mapping |

kdvs.core.GO.annotation.get\_term2probeset(*source\_db*, *go\_namespace*)¶
:   Get mapping of GO terms to GEDM probesets for requested GO namespace.

    |  |  |
    | --- | --- |
    | Parameters : | **source\_db** : db\_provider  tablespace that contains table ‘term2probeset’  **go\_namespace** : string  requested GO namespace |
    | Returns : | **term2probeset** : dict  dictionary that maps GO terms to sets of related probesets |

kdvs.core.GO.annotation.get\_term2size(*source\_db*, *go\_namespace*)¶
:   Get counts of associated probesets for all GO terms available for requested GO namespace.

    |  |  |
    | --- | --- |
    | Parameters : | **source\_db** : db\_provider  tablespace that contains table ‘term2probeset’  **go\_namespace** : string  requested GO namespace |
    | Returns : | **term2size** : iterable of (term, count) tuples  probeset counts for all GO terms available for requested GO namespace |

### Quick search


Enter search terms or a module, class or function name.

### Navigation

- index
- modules |
- modules |
- next |
- previous |
- KDVS 0.0.1-alpha documentation »
- KDVS API »

© Copyright 2010-2012, Grzegorz Zycinski, Salvatore Masecchia, Annalisa Barla.
Created using Sphinx 1.1.2.
