# Supplementary material for: Knowledge Driven Variable Selection (KDVS) – a new approach to enrichment analysis of gene signatures obtained from high–throughput data
Source: Source Code Biol Med. 2013 Jan 9;8:2. doi: 10.1186/1751-0473-8-2 (PMC3605163; doi:10.1186/1751-0473-8-2)
Supplement: Additional file 1 — Source code of KDVS. Format: ZIP. It contains the Python source code, the documentation, and the internal data files. [file 1751-0473-8-2-S1.zip › KDVS/doc/_build/html/doc-api/GO_subm.html]

kdvs.core.GO.subm — KDVS 0.0.1-alpha documentation


### Navigation

- index
- modules |
- modules |
- next |
- previous |
- KDVS 0.0.1-alpha documentation »
- KDVS API »

# kdvs.core.GO.subm¶

Provides functionality for extraction of GEDM submatrices and handling labels.

kdvs.core.GO.subm.get\_labels(*source\_db*, *samples\_list=None*, *ignore\_label\_val=None*)¶
:   Get mapping of samples to labels (if present).

    |  |  |
    | --- | --- |
    | Parameters : | **source\_db** : db\_provider  tablespace that contains table ‘LABELS’  **samples\_list** : iterable/None  list of samples; if present, then resulting mapping will be ordered according to this list; if None, then resulting mapping is presented in load order  **ignore\_label\_val** : integer/None  ignore the sample if associated label has this value; if None, then all labels are honored |
    | Returns : | **labels** : iterable of (sample, label) tuples  mapping of samples to labels |

kdvs.core.GO.subm.get\_subm\_for\_term(*source\_db*, *term2probeset*, *term*)¶
:   Get submatrix of GEDM values for requested GO term.

    |  |  |
    | --- | --- |
    | Parameters : | **source\_db** : db\_provider  tablespace that contains table ‘GEDM’  **term2probeset** : dict  mapping of GO terms to GEDM probesets  **term** : string  requested GO term |
    | Returns : | **submat** : iterable of iterables  list of rows of GEDM values  **probesets** : iterable  probesets associated with rows of values in submat |

    See also

    get\_term2probeset()

### Quick search


Enter search terms or a module, class or function name.

### Navigation

- index
- modules |
- modules |
- next |
- previous |
- KDVS 0.0.1-alpha documentation »
- KDVS API »

© Copyright 2010-2012, Grzegorz Zycinski, Salvatore Masecchia, Annalisa Barla.
Created using Sphinx 1.1.2.
