# Supplementary material for: Knowledge Driven Variable Selection (KDVS) – a new approach to enrichment analysis of gene signatures obtained from high–throughput data
Source: Source Code Biol Med. 2013 Jan 9;8:2. doi: 10.1186/1751-0473-8-2 (PMC3605163; doi:10.1186/1751-0473-8-2)
Supplement: Additional file 1 — Source code of KDVS. Format: ZIP. It contains the Python source code, the documentation, and the internal data files. [file 1751-0473-8-2-S1.zip › KDVS/doc/_build/html/doc-api/api.html]

KDVS API — KDVS 0.0.1-alpha documentation


### Navigation

- index
- modules |
- modules |
- next |
- previous |
- KDVS 0.0.1-alpha documentation »

# KDVS API¶

- kdvs.core.GO.GEDM
- kdvs.core.GO.GOTermTree
- kdvs.core.GO.GOTermTreeManip
- kdvs.core.GO.HGNC
- kdvs.core.GO.annotation
- kdvs.core.GO.subm
- kdvs.core.config
- kdvs.core.db
- kdvs.core.error
- kdvs.core.execenv
- kdvs.core.metadata
- kdvs.core.provider
- kdvs.core.rint
- kdvs.core.util

### Quick search


Enter search terms or a module, class or function name.

### Navigation

- index
- modules |
- modules |
- next |
- previous |
- KDVS 0.0.1-alpha documentation »

© Copyright 2010-2012, Grzegorz Zycinski, Salvatore Masecchia, Annalisa Barla.
Created using Sphinx 1.1.2.
