# Supplementary material for: Knowledge Driven Variable Selection (KDVS) – a new approach to enrichment analysis of gene signatures obtained from high–throughput data
Source: Source Code Biol Med. 2013 Jan 9;8:2. doi: 10.1186/1751-0473-8-2 (PMC3605163; doi:10.1186/1751-0473-8-2)
Supplement: Additional file 1 — Source code of KDVS. Format: ZIP. It contains the Python source code, the documentation, and the internal data files. [file 1751-0473-8-2-S1.zip › KDVS/doc/_build/html/doc-api/config.html]

kdvs.core.config — KDVS 0.0.1-alpha documentation


### Navigation

- index
- modules |
- modules |
- next |
- previous |
- KDVS 0.0.1-alpha documentation »
- KDVS API »

# kdvs.core.config¶

Provides configuration handling.

kdvs.core.config.evaluate\_cfg\_file(*cfg\_file\_path*, *ignore\_default\_cfg*, *output\_stream*)¶
:   Evaluate requested KDVS configuration file and return all configuration variables.

    |  |  |
    | --- | --- |
    | Parameters : | **cfg\_file\_path** : string  path to KDVS configuration file  **ignore\_default\_cfg** : bool  if True, the default configuration file will be ignored; otherwise, the default configuration file will be evaluated before and options will be merged  **output\_stream** : handle  output stream to direct output messages produced during evaluation |
    | Returns : | **final\_vars** : dict  dictionary of all configuration variables evaluated from configuration file |

kdvs.core.config.get\_default\_GO\_termdb\_release()¶
:   Return full path for default GO release RDF-XML file.

kdvs.core.config.get\_default\_R\_data\_root\_path()¶
:   Return full path for directory containing default R local environment.

kdvs.core.config.get\_default\_cfg\_file\_path(*relpath='config/default.cfg.py'*)¶
:   Return full path for default KDVS configuration file.

kdvs.core.config.get\_default\_internal\_data\_root\_path()¶
:   Return full path for directory with KDVS internal data.

kdvs.core.config.get\_default\_vis\_data\_root\_path()¶
:   Return full path for directory containing data used with visualization.

### Quick search


Enter search terms or a module, class or function name.

### Navigation

- index
- modules |
- modules |
- next |
- previous |
- KDVS 0.0.1-alpha documentation »
- KDVS API »

© Copyright 2010-2012, Grzegorz Zycinski, Salvatore Masecchia, Annalisa Barla.
Created using Sphinx 1.1.2.
