# Supplementary material for: Knowledge Driven Variable Selection (KDVS) – a new approach to enrichment analysis of gene signatures obtained from high–throughput data
Source: Source Code Biol Med. 2013 Jan 9;8:2. doi: 10.1186/1751-0473-8-2 (PMC3605163; doi:10.1186/1751-0473-8-2)
Supplement: Additional file 1 — Source code of KDVS. Format: ZIP. It contains the Python source code, the documentation, and the internal data files. [file 1751-0473-8-2-S1.zip › KDVS/doc/_build/html/doc-api/db.html]

kdvs.core.db — KDVS 0.0.1-alpha documentation


### Navigation

- index
- modules |
- modules |
- next |
- previous |
- KDVS 0.0.1-alpha documentation »
- KDVS API »

# kdvs.core.db¶

Provides layer for all DB operations performed by KDVS.

*class* kdvs.core.db.KDVSDB(*arbitrary\_data\_root=None*, *provider\_cfg=None*)¶
:   Bases: object

    General manager of all DB operations performed by KDVS. Currently configured
    specifically for SQLite3, it provides:

    > - handling of meta-database that contains information of all used single databases (dubbed tablespaces)
    > - handling of multiple tablespaces, including opening, closing, and copying/moving tables between them
    > - automatic data import from DSV files

    |  |  |
    | --- | --- |
    | Parameters : | **arbitrary\_data\_root** : string/None  path to directory containing all database objects managed by this manager instance; also, all new database objects will be created here; if None, default path ‘~/.kdvs/’ will be used  **provider\_cfg** : dict/None  configuration for specific DB provider; if None, default configuration for SQLite3 provider is used |

    close(*dbname=None*)¶
    :   Close DB operations handled by the manager.

        |  |  |
        | --- | --- |
        | Parameters : | **dbname** : string/None  name of specific tablespace to be closed; if None, all opened tablespaces handled by this manager will be closed |

    copy\_table(*src\_tmpdb\_id*, *dst\_tmpdb\_id*, *src\_table\_name*, *dst\_table\_name=None*, *move=False*, *replace=False*)¶
    :   Copy/move database table between two existing tablespaces.

        |  |  |
        | --- | --- |
        | Parameters : | **src\_tmpdb\_id** : string  name of tablespace containing table to be copied  **dst\_tmpdb\_id** : string  name of tablespace where table will be copied  **src\_table\_name** : string  name of the table to be copied  **dst\_table\_name** : string/None  name of the table to be copied, or None if the same as source table  **move** : bool  if True, the table will be moved completely from one tablespace to another  **replace** : bool  if True, the destination table will be replaced silently (without specific replace request, exception will be thrown if destination table already exists) |

    get\_tmpdb(*tmpdb\_id*)¶
    :   Return handle for requested tablespace; if tablespace does not exist, create it.

        |  |  |
        | --- | --- |
        | Parameters : | **tmpdb\_id** : string  name of requested tablespace |
        | Returns : | **handle** : db\_provider  handle for requested tablespace |

    get\_tmpdb\_loc(*tmpdb\_id*)¶
    :   Return path for file containing requested tablespace.

        |  |  |
        | --- | --- |
        | Parameters : | **tmpdb\_id** : string  name of requested tablespace |
        | Returns : | **location** : string  path to file that contains requested tablespace; None if tablespace does not exist, or such information is not available |

    load\_csv(*tmpdb\_id*, *descriptor*)¶
    :   Import data from DSV file to a table in requested tablespace, according to given DSV descriptor.

        |  |  |
        | --- | --- |
        | Parameters : | **tmpdb\_id** : string  name of tablespace where a table will be created  **descriptor** : iterable  descriptor containing the following data:  - absolute path of DSV file (string) - name of the destination table (string) - specific KDVS metadata for this DSV file (kdvs.core.metadata) |

kdvs.core.db.probeset2gene\_schema *= ('probeset2gene', 'probeset\_id', 'gene\_symbol', 'entrez\_id', 'gb\_acc')*¶
:   Default schema for probeset2gene table.

    See also

    create\_csv\_table\_st\_from\_schema()

kdvs.core.db.probeset2gene\_table *= 'probeset2gene'*¶
:   Name of probeset2gene table.

kdvs.core.db.term2probeset\_schema *= ('term2probeset', 'term\_id', 'probeset\_id', 'term\_evc', 'term\_desc', 'term\_ns')*¶
:   Default schema for term2probeset table.

    See also

    create\_csv\_table\_st\_from\_schema()

kdvs.core.db.term2probeset\_table *= 'term2probeset'*¶
:   Name of term2probeset table.

### Quick search


Enter search terms or a module, class or function name.

### Navigation

- index
- modules |
- modules |
- next |
- previous |
- KDVS 0.0.1-alpha documentation »
- KDVS API »

© Copyright 2010-2012, Grzegorz Zycinski, Salvatore Masecchia, Annalisa Barla.
Created using Sphinx 1.1.2.
