# Supplementary material for: Knowledge Driven Variable Selection (KDVS) – a new approach to enrichment analysis of gene signatures obtained from high–throughput data
Source: Source Code Biol Med. 2013 Jan 9;8:2. doi: 10.1186/1751-0473-8-2 (PMC3605163; doi:10.1186/1751-0473-8-2)
Supplement: Additional file 1 — Source code of KDVS. Format: ZIP. It contains the Python source code, the documentation, and the internal data files. [file 1751-0473-8-2-S1.zip › KDVS/doc/_build/html/doc-api/execenv.html]

kdvs.core.execenv — KDVS 0.0.1-alpha documentation


### Navigation

- index
- modules |
- modules |
- next |
- previous |
- KDVS 0.0.1-alpha documentation »
- KDVS API »

# kdvs.core.execenv¶

Provides core functionality for coordinated code execution, including control
of parallel processing.

## kdvs.core.execenv.execenv¶

Provides functionality of encapsulated execution environment for small dependent
computational tasks.

*class* kdvs.core.execenv.execenv.ExecEnv¶
:   Bases: object

    Implements basic execution environment, where set of actions (separated
    computational tasks) is executed in order, and common set of environment variables
    (environment state) is available for any action to store and retrieve data.

    add\_action(*action\_func*, *\*args*, *\*\*kwargs*)¶
    :   Add new action to the environment and schedule for execution. Actions are executed
        in the add order. Action is given as a function with its arguments.

        |  |  |
        | --- | --- |
        | Parameters : | **action\_func** : callable  function to be executed  **args** : iterable  any additional positional arguments passed to function  **kwargs** : dict  any additional keyworded arguments passed to function |

    add\_var(*varkey*, *var*, *replace=False*)¶
    :   Add new variable to execution environment, with possible replacement. Variables
        are managed in a dictionary, therefore all rules for adding items to standard
        dictionaries apply here as well.

        |  |  |
        | --- | --- |
        | Parameters : | **varkey** : object  hashable key for new variable  **var** : object  value for new variable  **replace** : bool  if True, already existing variable will be replaced |
        | Raises : | **ValueError** :  if variable already exists and replacement was not requested |

    clear\_actions()¶
    :   Clear all added actions.

    del\_var(*varkey*)¶
    :   Remove requested variable from execution environment. If variable does not exist,
        do nothing.

        |  |  |
        | --- | --- |
        | Parameters : | **varkey** : object  key of variable to be removed |

    execute()¶
    :   Execute all added actions so far in add order (FIFO). When any action throws
        an exception during its run, the whole execution is stopped and diagnostic information
        is returned.

        |  |  |
        | --- | --- |
        | Returns : | **diagnostic** : tuple/None  None if all actions were executed silently, otherwise the following information is returned:  - number of action that has thrown an exception - total number of actions to be executed - failed action details, as tuple (action\_func\_callable, args, kwargs) - thrown exception details, as tuple (exception instance, result of ‘sys.exc\_info’) - details of actions already executed before failed action, as iterable of tuples (action\_func\_callable, args, kwargs) - details of actions to be executed after failed action, as iterable of tuples (action\_func\_callable, args, kwargs) |

        See also

        sys.exc\_info()

    format\_action\_spec(*action\_spec*)¶
    :   Return textual representation of action.

        |  |  |
        | --- | --- |
        | Parameters : | **action\_spec** : tuple  (action\_func\_callable, args, kwargs) |
        | Returns : | **action\_repr** : string  textual representation of action |

    update\_vars(*vardict*)¶
    :   Add all variables from requested dictionary to execution environment. See
        add\_var() for details. Variables are added without replacement.

        |  |  |
        | --- | --- |
        | Parameters : | **vardict** : dict  dictionary of variables to be added |
        | Raises : | **ValueError** :  if one of new variables already exists (replacements are not requested here) |

    var(*varkey*)¶
    :   Retrieve value of variable present in execution environment.

        |  |  |
        | --- | --- |
        | Parameters : | **varkey** : object  key of variable to be retrieved |
        | Returns : | **var** : object  value of requested variable |
        | Raises : | **ValueError** :  if variable does not exist |

    varkeys()¶
    :   Retrieve keys of all existing variables present in execution environment.

        |  |  |
        | --- | --- |
        | Returns : | **varkeys** : iterable  keys of variables present in execution environment |

*class* kdvs.core.execenv.execenv.LoggedExecEnv(*env\_cfg*)¶
:   Bases: kdvs.core.execenv.execenv.ExecEnv

    Implements logged execution environment, where global logger is provided for
    any action to use, according to specified configuration. Logger must be initialized
    outside and passed in configuration variables. The logger is available
    through environment variable ‘logger‘.

    See also

    logging

    |  |  |
    | --- | --- |
    | Parameters : | **env\_cfg** : dict  configuration variables for logged execution environment; the following variables are recognized:  - ‘logger’ - instance of a logger that will be used as environment logger - ‘log\_path’ - path to log file - ‘log\_name’ - name of a logger - ‘log\_level’ - level of a logger |

    execute\_all()¶
    :   Execute all actions; if any action fails, log the diagnostic information.

        See also

        execute()

## kdvs.core.execenv.pplus\_env¶

Provides functionality of parallel execution environment, based on PPlus.

*class* kdvs.core.execenv.pplus\_env.PPlusExecEnv(*env\_cfg*)¶
:   Bases: kdvs.core.execenv.execenv.LoggedExecEnv

    Implements logged PPlus execution environment, according to specified
    configuration. The logger is available through environment variable ‘logger‘.
    PPlus connection is available through environment variable ‘pplus\_connection‘.

    See also

    logging

    |  |  |
    | --- | --- |
    | Parameters : | **env\_cfg** : dict  configuration variables for logged PPlus execution environment; the following variables are recognized:  - ‘logger’ - instance of a logger that will be used as environment logger - ‘log\_path’ - path to log file - ‘log\_name’ - name of a logger - ‘log\_level’ - level of a logger - ‘pplus\_debug\_mode’ - enable PPlus debug mode (True/False) |

    deserialize\_pzp\_from\_filekey(*filekey*)¶
    :   Deserialize object from requested PPlus remote file.

        |  |  |
        | --- | --- |
        | Parameters : | **filekey** : string  PPlus remote file identifier |
        | Returns : | **obj** : object  deserialized object |

    pprint\_to\_filekey(*filekey*, *obj*, *binary=True*)¶
    :   Serialize textual representation of given input object to PPlus remote file.
        The representation is produced using ‘pprint‘.

        |  |  |
        | --- | --- |
        | Parameters : | **filekey** : string  PPlus remote file identifier  **obj** : object  object to be serialized  **binary** : bool  if True, force remote file to be opened in binary mode |

        See also

        pprint

    serialize\_pzp\_to\_filekey(*filekey*, *obj*, *binary=True*)¶
    :   Serialize given input object as PPlus remote file.

        |  |  |
        | --- | --- |
        | Parameters : | **filekey** : string  PPlus remote file identifier  **obj** : object  object to be serialized  **binary** : bool  if True, force remote file to be opened in binary mode |

    serialize\_txt\_to\_filekey(*filekey*, *lines*, *binary=True*)¶
    :   Serialize given sequence of strings to PPlus remote file.

        |  |  |
        | --- | --- |
        | Parameters : | **filekey** : string  PPlus remote file identifier  **lines** : iterable  sequence of strings to be serialized  **binary** : bool  if True, force remote file to be opened in binary mode |

### Table Of Contents

- kdvs.core.execenv
  - kdvs.core.execenv.execenv
  - kdvs.core.execenv.pplus\_env

### Quick search


Enter search terms or a module, class or function name.

### Navigation

- index
- modules |
- modules |
- next |
- previous |
- KDVS 0.0.1-alpha documentation »
- KDVS API »

© Copyright 2010-2012, Grzegorz Zycinski, Salvatore Masecchia, Annalisa Barla.
Created using Sphinx 1.1.2.
