# Supplementary material for: Knowledge Driven Variable Selection (KDVS) – a new approach to enrichment analysis of gene signatures obtained from high–throughput data
Source: Source Code Biol Med. 2013 Jan 9;8:2. doi: 10.1186/1751-0473-8-2 (PMC3605163; doi:10.1186/1751-0473-8-2)
Supplement: Additional file 1 — Source code of KDVS. Format: ZIP. It contains the Python source code, the documentation, and the internal data files. [file 1751-0473-8-2-S1.zip › KDVS/doc/_build/html/doc-api/metadata.html]

kdvs.core.metadata — KDVS 0.0.1-alpha documentation


### Navigation

- index
- modules |
- modules |
- next |
- previous |
- KDVS 0.0.1-alpha documentation »
- KDVS API »

# kdvs.core.metadata¶

Provides SVS metadata for various file types and content.

## kdvs.core.metadata.annotation\_metadata¶

Provides KDVS metadata for handling DSV files with annotations.

## kdvs.core.metadata.experiment\_metadata¶

Provides KDVS metadata for handling DSV files with numeric-oriented data.

## kdvs.core.metadata.go\_metadata¶

Provides various metadata for Gene Ontology (GO).

### Table Of Contents

- kdvs.core.metadata
  - kdvs.core.metadata.annotation\_metadata
  - kdvs.core.metadata.experiment\_metadata
  - kdvs.core.metadata.go\_metadata

### Quick search


Enter search terms or a module, class or function name.

### Navigation

- index
- modules |
- modules |
- next |
- previous |
- KDVS 0.0.1-alpha documentation »
- KDVS API »

© Copyright 2010-2012, Grzegorz Zycinski, Salvatore Masecchia, Annalisa Barla.
Created using Sphinx 1.1.2.
