# Supplementary material for: Knowledge Driven Variable Selection (KDVS) – a new approach to enrichment analysis of gene signatures obtained from high–throughput data
Source: Source Code Biol Med. 2013 Jan 9;8:2. doi: 10.1186/1751-0473-8-2 (PMC3605163; doi:10.1186/1751-0473-8-2)
Supplement: Additional file 1 — Source code of KDVS. Format: ZIP. It contains the Python source code, the documentation, and the internal data files. [file 1751-0473-8-2-S1.zip › KDVS/doc/_build/html/doc-api/provider.html]

kdvs.core.provider — KDVS 0.0.1-alpha documentation


### Navigation

- index
- modules |
- modules |
- next |
- previous |
- KDVS 0.0.1-alpha documentation »
- KDVS API »

# kdvs.core.provider¶

Contains set of useful providers for various generic classes of objects handled by KDVS.

*class* kdvs.core.provider.KDVSMetadata¶
:   Bases: dict

    Abstract class for KDVS metadata instances.

*class* kdvs.core.provider.db\_provider(*conn\_func*, *\*args*)¶
:   Bases: object

    Abstract provider of Python DB API 2.0.

    See also

    **PEP 249**

    Establishes connection with DB using given connection function with specified arguments.

    |  |  |
    | --- | --- |
    | Parameters : | **conn\_func** : callable  function that provides ‘connect’ mechanism for specific DB  **args** : iterable  any positional arguments passed into connection function |

kdvs.core.provider.file\_provider(*filename*, *\*args*, *\*\*kwargs*)¶
:   Return opened file object suitable for use with context manager, regardless of
    file type. Provides transparent handling of compressed files.

    |  |  |
    | --- | --- |
    | Parameters : | **filename** : string  path to specific file  **args** : iterable  any positional arguments passed into opener function  **kwargs** : dictionary  any keyword arguments passed into opener function |
    | Returns : | **file\_object** : file  opened file object, suitable for use with context manager |

*class* kdvs.core.provider.fpBzip2File¶
:   Bases: bz2.BZ2File

    Wrapper class to allow opening bzip2-ed files with context manager.

*class* kdvs.core.provider.fpGzipFile(*filename=None*, *mode=None*, *compresslevel=9*, *fileobj=None*, *mtime=None*)¶
:   Bases: gzip.GzipFile

    Wrapper class to allow opening gzip-ed files with context manager.

    Constructor for the GzipFile class.

    At least one of fileobj and filename must be given a
    non-trivial value.

    The new class instance is based on fileobj, which can be a regular
    file, a StringIO object, or any other object which simulates a file.
    It defaults to None, in which case filename is opened to provide
    a file object.

    When fileobj is not None, the filename argument is only used to be
    included in the gzip file header, which may includes the original
    filename of the uncompressed file. It defaults to the filename of
    fileobj, if discernible; otherwise, it defaults to the empty string,
    and in this case the original filename is not included in the header.

    The mode argument can be any of ‘r’, ‘rb’, ‘a’, ‘ab’, ‘w’, or ‘wb’,
    depending on whether the file will be read or written. The default
    is the mode of fileobj if discernible; otherwise, the default is ‘rb’.
    Be aware that only the ‘rb’, ‘ab’, and ‘wb’ values should be used
    for cross-platform portability.

    The compresslevel argument is an integer from 1 to 9 controlling the
    level of compression; 1 is fastest and produces the least compression,
    and 9 is slowest and produces the most compression. The default is 9.

    The mtime argument is an optional numeric timestamp to be written
    to the stream when compressing. All gzip compressed streams
    are required to contain a timestamp. If omitted or None, the
    current time is used. This module ignores the timestamp when
    decompressing; however, some programs, such as gunzip, make use
    of it. The format of the timestamp is the same as that of the
    return value of time.time() and of the st\_mtime member of the
    object returned by os.stat().

*class* kdvs.core.provider.sqlite3\_db\_provider(*\*args*)¶
:   Bases: kdvs.core.provider.db\_provider

    DB provider for SQLite3.

    See also

    sqlite3

    Connect to SQLite3 DB according to specified arguments.

    |  |  |
    | --- | --- |
    | Parameters : | **args** : iterable  any positional arguments passed into connection function |

kdvs.core.provider.sqlite3\_provider\_cfg *= {'OperationalError': <class 'sqlite3.OperationalError'>, 'provider': <class 'kdvs.core.provider.sqlite3\_db\_provider'>}*¶
:   Configuration parameters for SQLite3 DB provider.

### Quick search


Enter search terms or a module, class or function name.

### Navigation

- index
- modules |
- modules |
- next |
- previous |
- KDVS 0.0.1-alpha documentation »
- KDVS API »

© Copyright 2010-2012, Grzegorz Zycinski, Salvatore Masecchia, Annalisa Barla.
Created using Sphinx 1.1.2.
