# Supplementary material for: Knowledge Driven Variable Selection (KDVS) – a new approach to enrichment analysis of gene signatures obtained from high–throughput data
Source: Source Code Biol Med. 2013 Jan 9;8:2. doi: 10.1186/1751-0473-8-2 (PMC3605163; doi:10.1186/1751-0473-8-2)
Supplement: Additional file 1 — Source code of KDVS. Format: ZIP. It contains the Python source code, the documentation, and the internal data files. [file 1751-0473-8-2-S1.zip › KDVS/doc/_build/html/doc-api/rint.html]

kdvs.core.rint — KDVS 0.0.1-alpha documentation


### Navigation

- index
- modules |
- modules |
- next |
- previous |
- KDVS 0.0.1-alpha documentation »
- KDVS API »

# kdvs.core.rint¶

Provides layer for integration with R environment.

kdvs.core.rint.R()¶
:   Return global R interpreter instance.

kdvs.core.rint.Rcall(*r\_code\_str*, *silent=True*)¶
:   Execute given R statement(s) in the context of current R environment.

    |  |  |
    | --- | --- |
    | Parameters : | **r\_code\_str** : string  R statement(s) to be executed  **silent** : bool  If True, all R messages produced during execution will be suppressed. If False, they will be shown on standard output. |
    | Returns : | **res** : object  any result returned from execution of R statement(s), in form of R object(s) |

    See also

    rpy2.robjects

kdvs.core.rint.Rimport(*r\_package\_name*, *silent=True*)¶
:   Import given R package “as is” in the context of current R environment. For more
    control over import process, use ‘importr’ directly.

    |  |  |
    | --- | --- |
    | Parameters : | **r\_package\_name** : string  name of R package to be imported  **silent** : bool  If True, all R messages produced during import will be suppressed. If False, they will be shown on standard output. |
    | Returns : | **rpkg** : rpy2.robjects.packages.Package  instance of R imported package |

    See also

    rpy2.robjects.packages.importr()

kdvs.core.rint.Robj()¶
:   Return global R environment, including all R objects available through it.

    See also

    rpy2.robjects

### Quick search


Enter search terms or a module, class or function name.

### Navigation

- index
- modules |
- modules |
- next |
- previous |
- KDVS 0.0.1-alpha documentation »
- KDVS API »

© Copyright 2010-2012, Grzegorz Zycinski, Salvatore Masecchia, Annalisa Barla.
Created using Sphinx 1.1.2.
