# Supplementary material for: Knowledge Driven Variable Selection (KDVS) – a new approach to enrichment analysis of gene signatures obtained from high–throughput data
Source: Source Code Biol Med. 2013 Jan 9;8:2. doi: 10.1186/1751-0473-8-2 (PMC3605163; doi:10.1186/1751-0473-8-2)
Supplement: Additional file 1 — Source code of KDVS. Format: ZIP. It contains the Python source code, the documentation, and the internal data files. [file 1751-0473-8-2-S1.zip › KDVS/doc/_build/html/doc-api/util.html]

kdvs.core.util — KDVS 0.0.1-alpha documentation


### Navigation

- index
- modules |
- modules |
- previous |
- KDVS 0.0.1-alpha documentation »
- KDVS API »

# kdvs.core.util¶

*class* kdvs.core.util.NullOutputStream¶
:   Bases: object

    Emulates stream that ignores all output sent into it, for compatibility with Python 2.6.

kdvs.core.util.create\_csv\_table\_st\_from\_schema(*schema\_components*, *default\_col\_type='TEXT'*)¶
:   Make SQL ‘create table’ statement for tables storing data imported from DSV files.

    |  |  |
    | --- | --- |
    | Parameters : | **schema\_components** : iterable  schema of table to be created  **default\_col\_type** : string  common type of all columns for table to be created |
    | Returns : | **statement** : string  string containing SQL statement that creates the table |

kdvs.core.util.find\_str\_in\_seq(*seq*, *pattern*)¶
:   Check if any string in sequence contains pattern.

    |  |  |
    | --- | --- |
    | Parameters : | **seq** : iterable  sequence of strings  **pattern** : string  pattern to find |
    | Returns : | **found** : bool  True if pattern found in sequence |

kdvs.core.util.is\_metadata(*dic*)¶
:   Check if given dictionary is proper KDVS metadata dictionary.

    |  |  |
    | --- | --- |
    | Parameters : | **dic** : KDVSMetadata/dict  dictionary to be checked |
    | Returns : | **isMetadata** : bool  True if dictionary id proper KDVS metadata dictionary |

kdvs.core.util.pzp\_deserialize(*obj\_path*)¶
:   Deserialize object from file using PZP protocol.

    |  |  |
    | --- | --- |
    | Parameters : | **obj\_path** : string  path to file containing object to deserialize |
    | Returns : | **obj** : object  deserialized object |

kdvs.core.util.pzp\_deserialize\_obj(*encoded\_object*)¶
:   Deserialize given encoded object using PZP protocol.

    |  |  |
    | --- | --- |
    | Parameters : | **encoded\_object** : string  string containing serialized object |
    | Returns : | **obj** : object  de-serialized object |

kdvs.core.util.pzp\_serialize(*input\_obj*, *obj\_path*)¶
:   Serialize given input object using PZP protocol to file.

    |  |  |
    | --- | --- |
    | Parameters : | **input\_object** : object  object to be serialized  **obj\_path** : string  path to file where serialized object will be stored |

kdvs.core.util.pzp\_serialize\_obj(*input\_object*)¶
:   Serialize given input object using PZP protocol to string.

    |  |  |
    | --- | --- |
    | Parameters : | **input\_object** : object  object to be serialized |
    | Returns : | **serialized\_obj** : string  string containing serialized object |

kdvs.core.util.quote(*s*, *quote='"'*)¶
:   Surrounds requested string with given “quote” strings.

    |  |  |
    | --- | --- |
    | Parameters : | **s** : string  string to quote  **quote** : string  string to be appended to the beginning and the end of requested string |
    | Returns : | **quoted\_str** : string  quote + s + quote |

kdvs.core.util.skip\_comment\_reader(*seq*, *comment\_string='#'*)¶
:   Generator that discards strings prefixed with given comment string.

    |  |  |
    | --- | --- |
    | Parameters : | **seq** : iterable  sequence of strings  **comment\_string** : string  comment prefix |
    | Returns : | **ncomseq** : iterable  filtered input sequence of strings without commented ones |

kdvs.core.util.sniff\_dsv(*dsvobj*, *sample\_lines\_count=10*)¶
:   Sniff opened DSV content, given either as file or sequence/generator of lines,
    by reading given number of lines from the beginning as a sample.

    |  |  |
    | --- | --- |
    | Parameters : | **dsvobj** : file handle/sequence/generator  DSV content, either as opened file handle or sequence/generator of lines  **sample\_lines\_count** : integer  number of lines to be read during sniffing |
    | Returns : | **sniffed\_dialect** : csv.Dialect  sniffed csv.Dialect instance  **peek\_lines** : iterable  sequence of lines read during sniffing; sample lines are not returned to the content automatically and must be appended manually, e.g.:  ``` # f is file/sequence/generator of lines dialect, peek_lines = sniff_dsv(f) # return sniffed lines back to the content import itertools f = itertools.chain(peek_lines, f) ``` |

### Quick search


Enter search terms or a module, class or function name.

### Navigation

- index
- modules |
- modules |
- previous |
- KDVS 0.0.1-alpha documentation »
- KDVS API »

© Copyright 2010-2012, Grzegorz Zycinski, Salvatore Masecchia, Annalisa Barla.
Created using Sphinx 1.1.2.
