# Supplementary material for: Knowledge Driven Variable Selection (KDVS) – a new approach to enrichment analysis of gene signatures obtained from high–throughput data
Source: Source Code Biol Med. 2013 Jan 9;8:2. doi: 10.1186/1751-0473-8-2 (PMC3605163; doi:10.1186/1751-0473-8-2)
Supplement: Additional file 1 — Source code of KDVS. Format: ZIP. It contains the Python source code, the documentation, and the internal data files. [file 1751-0473-8-2-S1.zip › KDVS/doc/_build/html/doc-gen/additional.html]

Additional topics — KDVS 0.0.1-alpha documentation


### Navigation

- index
- modules |
- modules |
- next |
- previous |
- KDVS 0.0.1-alpha documentation »

# Additional topics¶

## KDVS Execution Model¶

### Overview¶

KDVS applications are based on the principle of execution environment, an
entity that provides all resources needed by the code to complete requested
task. The environment is composed of actions and environment variables.

Environment simply executes series of actions added in specified order:

```
env=prepare_env()
env.add_action(action1)
env.add_action(action2)
# ...
env.add_action(actionN)
# execute action1, action2, ..., actionN in order
env.execute()
```

Actions are autonomous units of code that perform requested tasks. Here,
they are simply function objects:

```
def action_func(env):
    # ...
    # do something
    # ...
    pass    #optional

# add action to the environment
# ...
env.add_action(action_func)
# ...
```

Actions can have additional arguments if needed:

```
def action_func(env, arg1, arg2):
    # ...
    # do something with arg1 and arg2
    # ...
    pass    #optional

# add action to the environment
# ...
env.add_action(action_func, arg1_value, arg2_value)
# ...
```

Currently, only explicit functions are supported; automated import of serialized
function objects is planned.

If an action produces results that need to be shared with another actions that
run later, it can use environment variables to store them:

```
# store new environment variable for later actions to use:
env.add_var('new_variable_name', new_variable_value)
```

Analogically, any action can get environment variable that was created earlier:

```
# use existing environment variable from earlier actions:
variable = env.var('variable_name')
```

Currently, if requested environment variable is not available, the action must
handle this case by itself (but see Exception handling); support for variable
checking and verification is planned.

### Exception handling¶

If at least one exception is thrown from within any action, the whole execution
flow of the environment stops, and the diagnostic information is shown or logged:

- number of action that has thrown an exception
- total number of actions to be executed
- failed action details, namely action name and any additional action arguments (excluding ‘env’)
- thrown exception details, as exception name and arguments, and available stack trace
- details of actions already executed before failed action, in the same format as
  failed action details
- details of actions to be executed after failed action, in the same format as
  failed action details

Note

Actions are supposed *not to* throw any exceptions outside its function
body. All execution must be self-contained, and actions are allowed to transfer
its state only through environment variables; deliberate exception propagation
will lead to error.

Currently, the environment variables created before exception was thrown,
are **not** preserved after execution flow has stopped; support for preservation
of environment is planned.

### Preparing environment¶

When creating requested execution environment and initializing it with specific
variables if needed, KDVS applications, by convention, use dedicated function
‘prepare\_env’:

```
def prepare_env():
    # ...
    # ...
    return env
```

It must return fully initialized instance of ExecEnv or one of its subclasses.

Note

In case of logged environment, the logger itself must be created and
initialized within this function, and any diagnostic messages from
activities *before that point* will not be available. This may be
circumvented by capturing early diagnostic messages into StringIO
stream and log them just before environment instance is returned.

### Configuring environment¶

Beside manual environment configuration, any KDVS application can utilize
configuration files for importing batch of variables at once, using
evaluate\_cfg\_file() function from the core API.

The configuration file is normal Python script that contains Python statements
to be executed. It is opened and parsed using execfile() mechanism. The
file may contain any valid Python code, including import statements if
needed. It must contain syntactically correct sequence of statements; see
execfile() for more details.

Every assignment of the form:

```
var_name = var_value
```

is captured as new variable ‘var\_name’ with value ‘var\_value’. In other words,
every local variable evaluated inside configuration file is captured.

As a result, evaluate\_cfg\_file() returns dictionary of variables
evaluated from specified configuration file.

Note

- The configuration file may contain **any** valid Python code to be executed;
  do not use configuration file from unsafe source
- Since the configuration file is normal Python script, it may be checked for
  errors before use by executing it with Python interpreter
- When specifying variables with string value, the value must be enclosed
  either within single or double quotes:

  ```
  option1 = 'simplevalue'
  option2 = "value with some spaces"
  ```
- When specifying variables with list/tuple/dict value, the value must conform
  to the standard Python syntax:

  ```
  # variable with list value, 3 elements
  var_list = ['val1', 'val2', 'val3']
  # variable with tuple value, 3 elements
  var_tuple1 = ('val1', 'val2', 'val3')
  var_tuple2 = 'val1', 'val2', 'val3'  # equivalent
  # variable with tuple value, 1 element
  var_tuple3 = ('val1',)
  var_tuple4 = 'val1',     # equivalent
  # variable with dictionary value, 3 key-value pairs
  var_dict = {'key1' : 'val1', 'key2' : 'val2', 'key3' : 'val3'}
  ```

#### Default configuration file¶

The location of default configuration file is available to any KDVS application by
get\_default\_cfg\_file\_path().

In typical scenario, default configuration file is evaluated at the beginning
of KDVS application life cycle, and user specified configuration file is evaluated
afterwards, effectively overwriting default variables:

```
from kdvs.core.config import evaluate_cfg_file
import cStringIO
# ...
# stream for capturing any diagnostic messages
output_stream=cStringIO.StringIO()
# evaluate default configuration file first, and user configuration file afterwards
config_vars = evaluate_cfg_file('path/to/user.cfg.py', ignore_default_cfg=False, output_stream)
# create simple environment instance
env = ExecEnv()
env.update_vars(config_vars)
# ...
```

See evaluate\_cfg\_file() for specific details.

## Parsing metadata¶

In order for KDVS to use *Input Data*, it must be known how to parse
it and make it useful. To do so, KDVS introduces one more layer of metadata called
“parsing metadata”. It provides information how to parse files containing
raw numerical data and annotation metadata.

Parsing metadata is coming in the form of specific key-value pairs in Python
dictionaries that describe particular raw data and annotation metadata. Those
dictionaries are passed to appropriate methods and functions that parses raw
data/annotation metadata files.

Below is the complete example of parsing metadata; it is used to parse default
raw DSV files containing numerical data:

```
{
    # ---- metadata regarding file structure
    'dialect' : 'sniff',
    'comment_prefix' : None,
    # ---- metadata regarding file content
}
```

### File structure metadata¶

This metadata organizes information regarding physical parsing of files. Since
DSV files of both types mostly have the similar structure, it is possible to
define parsing strategy for the most popular cases. Also, when more elaborated
format is used, more specific strategy can be presented.

The following key-value pairs are recognized as part of file structure metadata:

> - dialect : excel/excel-tab/sniff/custom
>
>   Specifies which format to parse.
>
>   - excel
>
>     The file is in CSV format (comma-separated values).
>   - excel-tab
>
>     The file is in TSV format (tabulator-separated values).
>   - sniff
>
>     If we are not sure which DSV case exactly we are dealing with, it is
>     possible to guess it using heuristics; however, there is no 100%
>     guarantee that the guess is correct.
>   - custom
>
>     When dealing with non-DSV files, the parsing strategy may be hard-coded
>     and presented in the form of regular expressions to use, bypassing standard
>     DSV parsing strategy; such parsing is considerably slower, but it enables
>     to use sometimes more elaborated annotation metadata.
> - comment\_prefix : None/"<prefix\_string>"/'<prefix\_string>'
>
>   Sometimes, the DSV file may contain commented lines that should be
>   discarded (they are present only for human readability); the most common
>   case is that commented line contains special prefix string to enable fast
>   identification.
>
>   - None
>
>     There are no commented lines in that file.
>   - "<prefix\_string>"/'<prefix\_string>'
>
>     There are commented lines prefixed with “prefix” string. For example:
>
>     ```
>     # Created on Jun 4, 2010
>     # Author Grzegorz Zycinski
>     "Row ID","Column1","Column2","Column3"
>     "Row1",1.00,2.00,3.00
>     "Row2",4.00,5.00,6.00
>     ```
>
>     In this case, first two lines are comment lines and should be discarded;
>     the comment prefix is “#” (without trailing whitespaces).

### File content metadata¶

This metadata organizes information that we want to retrieve from numerical
data/annotation metadata files. It may be expressed in a number of ways,
according to the particular task.

1. For numerical data, we are usually interested in the set of values in subsequent
   rows. Since this is the default strategy, we do not need to specify it.
   For example, in the default parsing rules of numerical data, this subsection
   is empty:

   ```
   NUM_DSV_METADATA=KDVSMetadata({
       # ---- metadata regarding file structure
       # csv 'dialect' used to parse this particular file
       # if 'sniff' then use csv sniffer to determine proper file parsing
       'dialect' : 'sniff',
       # file may contain commented lines
       # comment prefix or None if file contains no commented lines
       'comment_prefix' : None,
       # ---- metadata regarding file content
   })
   ```
2. In the case of parsing annotation metadata in standard DSV file, we are
   often interested in the information stored in particular columns of the
   file. The typical way is to specify a set of columns we are interested in
   along with their unique identifiers in the file. Also, if we expect
   particular values in the file, we can indicate them as well.

   For example, we are interested in extracting 4 specific columns from annotation
   data file, namely ID, Gene Ontology Biological Process,
   Gene Ontology Cellular Component, and Gene Ontology Molecular Function,
   and we can expect some particular values appearing in last 3 columns. Each
   column has its unique identifier, along with the expected values. This may
   be expressed as follows:

   ```
   ANNOFILE_METADATA_GEO_GPL=KDVSMetadata({
       # ---- metadata regarding file structure
       # ... file structure metadata goes here ...
       # ---- metadata regarding file content
       # column aliases
       'probeset_id_col' : 'ID',
       'BP' : 'Gene Ontology Biological Process',
       'CC' : 'Gene Ontology Cellular Component',
       'MF' : 'Gene Ontology Molecular Function',
       'sequence_type' : 'Sequence Type',
       'gene_symbol' : 'Gene Symbol',
       'gb_acc' : 'GB_ACC',
       # what symbol is used in annotation file when there are no GO terms
       # associated with particular probe
       'go_terms_absent_for_probe' : '',
       # separator for different GO terms assigned to the same probe
       'go_terms_separator' : '///',
       # separator for different parts of every GO term parsed
       'go_term_part_separator' : '//',
       # tag for control sequence
       'tag_control_seq' : 'Control sequence',
       # separator for different gene symbols
       'gene_symbol_separator' : '///',
   })
   ```

   We can pass these rules to the parsing code that will recognize those
   identifiers and will use them to process DSV file accordingly.
3. In the case of parsing annotation metadata in custom format, we are interested
   both in successful parsing and extracting valuable information. In that case,
   we should always specify dialect as custom, and file content
   metadata will contain two sub-sections, description part and parsing part.

   Description part is very similar to the one described in (2) and plays
   similar role. Parsing part contains additional information for parser, i.e.
   how single line is constructed, and what fields we want to focus on.

   Typically, the following key-value pairs are recognized:

   - line\_patt\_str : <regexp\_string>

     Specifies complete
     regular expression
     used to parse a single line of this file. See re for more details.
   - matches : iterable of strings

     Specifies which parts of the line we want to capture; the strings themselves
     may be used as unique field identifiers for further processing; the indexes
     of the strings in the iterable must match the groups captured in regular
     expression.

     For instance, in the following regular expression we are capturing three
     groups (see
     syntax reference
     for more details) with round brackets ():

     ```
     '[^\:]+\:(\d+)\s+([^\:]+)\:(\w+)\s*'
     ```

     Now, we can identify those three fields with corresponding three matches:

     ```
     ('gene_id', 'kegg_db_id', 'kegg_db_entity_id')
     ```

   Below is the complete example of metadata that can be used to parse one of the
   exotic annotation metadata, EXT2GO:

   ```
   ANNOFILE_METADATA_EXT2GO=KDVSMetadata({
       # ---- metadata regarding file structure
       'dialect' : 'custom',
       # file may contain commented lines
       # comment prefix or None if file contains no commented lines
       'comment_prefix' : '!',
       # ---- metadata regarding file content
       # -- description part
       # prefix of every GO related entity in EXT2GO
       'go_id' : 'GO:',
       # separator between formal EXT DB id and entity id, e.g. KEGG:R00004
       'db_id_sep' : ':',
       # separator between mapping parts
       'map_sep' : '>',
       # separator between GO name part and term part
       'term_desc_name_sep' : ';',
       # -- parsing part
       # regexp for parsing single line of EXT2GO
       # the following fields are captured:
       # - ID of external DB
       # - entity ID in external DB
       # - entity description if provided
       # - name of corresponding GO term
       # - ID of corresponding GO term
       'line_patt_str' : '(\w+)\%s(\w+)\s*(.*)\s+\%s\s+%s([^\%s]+)\s+\%s\s+%s(\d+)'%(':', '>', 'GO:', ';', ';', 'GO:'),
       # field indexes in regexp match (may be directly used as column names)
       'matches' : ('db_id', 'entity_id', 'entity_desc', 'term_desc', 'term_id'),
   })
   ```

Note

Currently KDVS does not use metadata in custom format. The corresponding
parsing functionality is treated as **experimental**.

Note

In the case of file structure metadata, it is possible to define more or
less coherent set of basic rules, that may be used as template. As a result,
the parsing code that interpretes them is general.

However, in the case of file content metadata, there are no stringent rules
for specifying it. This portion of parsing metadata is tailored for specific
needs, and is closely associated with the specific parsing code that uses them.
Therefore, file content metadata should be defined and used with caution.

## PZP¶

### Overview¶

PZP storage protocol was devised as standard method for storing data by KDVS.

Since majority of input information comes in the form of plain text, and also
there is much intermediate information to be transferred over local network,
there was a need for a method that is simple enough and offers acceptable
compression level, as well as some basic verification mechanisms, when applied.

All data that need to be transported across network, or stored for further
reference, unless specified, are stored in PZP format. By convention, shared
with PPlus, the files produced by KDVS that contain PZP content have capitalized
names, e.g. CFG, TERM\_2\_SIZE etc.

KDVS contains simple application *pzp\_dump* that provides
textual representation of PZP content.

### Details¶

The PZP (Pickle, Zip, Pickle) protocol operates on Python objects as follows:

- pickle the selected Python object with lowest possible pickle protocol
  (currently 0) into string (*pass1*)
- compress resulting string, using zlib.compress() with default parameters
  (*pass2*)
- pickle zipped string again with highest possible pickle protocol
  (currently pickle.HIGHEST\_PROTOCOL) into string or file (*pass3*)

Pickling in pass1 generates verbose representation that can be zipped
effectively in pass2; pickling again in pass3, while seemingly redundant,
provides simple and convenient mechanism of data verification when restoring
original object, all handled completely by Python standard library in few lines:

```
def pzp_serialize(input_object):
    import cPickle
    import zlib
    # pickle, zip, pickle
    pass1=cPickle.dumps(input_object, protocol=0)
    pass2=zlib.compress(pass1)
    pass3=cPickle.dumps(pass2, protocol=cPickle.HIGHEST_PROTOCOL)
    return pass3
```

Analogically, reciprocal UUU (Unpickle, Unzip, Unpickle) protocol provides
mechanism for restoring original object, again in few lines:

- un-pickle given input PZP string (*pass1*)
- un-zip resulting string (*pass2*)
- un-pickle resulting string again (*pass3*)

```
def pzp_deserialize(encoded_object):
    import cPickle
    import zlib
    # unpickle, unzip, unpickle
    pass1=cPickle.loads(encoded_object)
    pass2=zlib.decompress(pass1)
    pass3=cPickle.loads(pass2)
    return pass3
```

Note

Using PZP on large Python objects is both memory and time consuming.

## PPlus¶

While l1l2 is a
powerful statistical learning technique, it consumes a lot of computational
resources. However, it is easy to parallelize its execution based on the
concepts of external splits (see
L1L2Py documentation and
references therein).

KDVS relies heavily on frequent use of l1l2. To ease computational burden
related to this fact, the PPlus library
was created. It provides simple infrastructure for running computationally-heavy,
data-rich numerical experiments implemented in Python, using parallelization.
*experiment* application was designed to utilize PPlus.

See PPlus documentation for more details.

### Table Of Contents

- Additional topics
  - KDVS Execution Model
    - Overview
    - Exception handling
    - Preparing environment
    - Configuring environment
      - Default configuration file
  - Parsing metadata
    - File structure metadata
    - File content metadata
  - PZP
    - Overview
    - Details
  - PPlus

### Quick search


Enter search terms or a module, class or function name.

### Navigation

- index
- modules |
- modules |
- next |
- previous |
- KDVS 0.0.1-alpha documentation »

© Copyright 2010-2012, Grzegorz Zycinski, Salvatore Masecchia, Annalisa Barla.
Created using Sphinx 1.1.2.
