# Supplementary material for: Knowledge Driven Variable Selection (KDVS) – a new approach to enrichment analysis of gene signatures obtained from high–throughput data
Source: Source Code Biol Med. 2013 Jan 9;8:2. doi: 10.1186/1751-0473-8-2 (PMC3605163; doi:10.1186/1751-0473-8-2)
Supplement: Additional file 1 — Source code of KDVS. Format: ZIP. It contains the Python source code, the documentation, and the internal data files. [file 1751-0473-8-2-S1.zip › KDVS/doc/_build/html/doc-gen/applications.html]

Applications — KDVS 0.0.1-alpha documentation


### Navigation

- index
- modules |
- modules |
- next |
- previous |
- KDVS 0.0.1-alpha documentation »

# Applications¶

## experiment¶

Implements the experimental part of prototype framework for devising gene
signatures from microarray data while applying prior knowledge from Gene
Ontology. The framework utilizes regularization approach implemented in L1L2Py,
executed in parallel environment controlled by PPlus.

See *Methodology* for descriptive characteristics of the framework, as
well as for statistical concepts behind it.

It works as standalone command line application, with the following options:

|  |  |
| --- | --- |
| `-c CFGFILE` | read configuration from CFGFILE |
| `-t TDIR` | store temporary database(s) in TDIR |
| `-l LOGFILE` | direct log output to LOGFILE (with default log level INFO) |
| `--ignore-default-config` | |
|  | ignore default configuration file |
| `--test-mode` | run in test mode (only fraction of computational jobs will be executed) |
| `--pplus-debug-mode` | |
|  | use PPlus in debug mode (see PPlus integration for details) |
| `--debug` | set log level to DEBUG |

### Configuration file¶

The configuration file is the central resource for experiment. It should
provide all the variables needed to complete the statistical experiment.

Currently, the following variables are recognized:

| Name | Type | Description |
| --- | --- | --- |
| **General** | | |
| task\_name | string | readable description of the experiment; for information purposes only |
| **Experiment Input** | | |
| gedm\_data\_file | string | path to DSV file that contains normalized GEDM (gene expression data matrix) numerical data; the file must be row-oriented (each row contains expression values for specific probeset across all samples considered) |
| annotation\_file | string | path to DSV file that contains annotations for probesets listed in GEDM |
| go\_namespaces | tuple | symbols of Gene Ontology namespaces to consider; possible values: ‘BP’, ‘MF’, ‘CC’ |
| labels\_file | string/None | path to DSV file containing labels, or None if not used |
| **Experiment Output** | | |
| result\_dir | string | path to output directory where the results will be stored |
| transfer\_disk\_data | boolean | transfer all the data from remote disk after job results are collected |
| **Experiment Parameters** | | |
| experiment\_type | string | type of l1l2 experiment to execute, proper error functions will be determined automatically; to override default settings, see ‘L1L2Py advanced configuration’ section; possible values: ‘classification’, ‘balanced\_classification’, ‘regression’ |
| submatrices\_rows\_threshold | integer | threshold for number of rows in submatrices; below this value, RLS/OLS is used; above this value, full model selection is used |
| ridge\_regression\_option | string | run either RLS or OLS (lambda=0.0); possible values: ‘RLS’, ‘OLS’; default is ‘OLS’ |
| tau\_min\_scale | float | minimum scaling factor for l1l2 tau |
| tau\_max\_scale | float | maximum scaling factor for l1l2 tau |
| tau\_range\_type | string | type of range of l1l2 tau values to be used; possible values: ‘geometric’, ‘linear’ |
| tau\_number | integer | number of elements for l1l2 tau range of elements |
| lambda\_min | float | minimum value for l1l2 lambda |
| lambda\_max | float | maximum value for l1l2 lambda |
| lambda\_range\_type | string | type of range of l1l2 lambda values to be used; possible values: ‘geometric’, ‘linear’ |
| lambda\_number | integer | number of elements for l1l2 tau range of elements |
| lambda\_range | tuple | direct specification of range of l1l2 lambda values; when used, it will override automatically generated l1l2 lamdba range |
| mu\_scaling\_factor\_min | float | minimum scaling factor for l1l2 mu |
| mu\_scaling\_factor\_max | float | maximum scaling factor for l1l2 mu |
| mu\_range\_type | string | type of range of l1l2 mu values to be used; possible values: ‘geometric’, ‘linear’ |
| mu\_number | integer | number of elements for l1l2 mu range of elements |
| external\_k | integer | number of cross validation steps in external loop of l1l2 model selection |
| internal\_k | integer | number of cross validation steps in internal loop of l1l2 model selection |
| sparse | boolean | prefer sparsest solution in stage I of l1l2 model selection; default is False (exclusive with ‘regularized’) |
| regularized | boolean | prefer most regularized solution in stage I of l1l2 model selection; default is True (exclusive with ‘sparse’) |
| return\_predictions | boolean | for classification type of experiments, return predicted labels along with prediction errors; default is True |
| threshold\_error | float | threshold for l1l2 average test error calculated for single submatrix; when below, the experiment results for this submatrix will be accepted |
| threshold\_frequency | float | threshold for frequency of variables appearing in nested lists; when below, the variable will be skipped |
| ignore\_label\_val | integer | value for any labels to be excluded; all data related to samples associated with those labels will not be considered |
| **L1L2Py advanced configuration** | | |
| cv\_error\_func | callable | function that calculates cross validation error in l1l2 stage I according to requested experiment type; normally it is selected automatically based on experiment\_type; see example configuration file for details |
| error\_func | callable | function that calculates training and test error in l1l2 stage II according to requested experiment type; normally it is selected automatically based on experiment\_type; see example configuration file for details in l1l2 experiment; 0 by default |
| data\_normalizer | callable | function for normalizing numerical matrices processed during l1l2 experiment; see example configuration file for details |
| labels\_normalizer | callable | function for normalizing label vectors processed during classification-type l1l2 experiment; see example configuration file for details |
| **PPlus advanced configuration** | | |
| cfg\_filekey | string | filekey for serialized object that holds initial experiment configuration; default is CFG |
| txt\_suffix | string | name suffix for files that contain textual representation of certain objects; default is ‘.txt’ |
| term2size\_filekey | string | filekey for serialized object that holds term2size mapping (see get\_term2size() for details); default is TERM\_2\_SIZE |
| samples\_list\_filekey | string | filekey for serialized object that holds the list of all samples as loaded from GEDM data; default is SAMPLES\_LIST |
| probesets\_list\_filekey | string | filekey for serialized object that holds the list of all probesets as loaded from GEDM data; default is PROBESETS\_LIST |
| labels\_ref\_filekey | string | filekey for textual representation of labels as loaded from label data; default is LABELS |
| labels\_mat\_filekey | string | filekey for numerical vector of labels, if any, used in l1l2 experiment; default is LABELS\_MAT |
| submatrix\_filekey\_suffix | string | name suffix for files that contain submatrix data for associated GO term; default is \_\_ (two underscores) |
| submatrix\_reading\_list\_suffix | string | name suffix for serialized objects that hold lists of GO terms grouped under specific conditions; default is \_RL |
| probeset2geneid\_filekey | string | filekey for serialized object that holds probeset2geneid mapping (see get\_probeset2geneid() for details); default is PROBESET\_2\_GENEID |
| disk\_data\_suffix | string | directory suffix appended to data directory transferred from remote disk; default is \_disk\_data |
| transfer\_disk\_data\_logs | boolean | preserve individual job logs when transferring data directory from remote disk; default is True |

### Environment¶

#### Overview¶

experiment uses PPlusExecEnv, configured to share central remote
disk across dedicated group of machines available in local network. The machine
that runs experiment application is experiment master, the remaining
ones are experiment workers. Experiment master controls all temporary database(s)
that are created during execution, and collects all remote data.

All time-consuming computations, namely atomic l1l2 experiments, are distributed
as jobs controlled by PPlus, across all the dedicated machines, and results are
transferred to shared remote disk, to common ‘experiment directory’. After all jobs are
finished, the content of ‘experiment directory’ is transferred back to experiment
master. At the end, experiment master machine contains all the results in result\_dir
directory.

#### prepare\_env()¶

prepare\_env() function performs set of the following activities (when
referring to particular environment variable, $var\_name is the value of
variable identified by var\_name):

- Process command line arguments and set option values.
- Locate user configuration file.

  The file must be present on experiment master machine filesystem. All output
  data will be located in the directory that contains user config file, e.g. if
  the config file path is /usr/local/kdvs/user.cfg, then all output data and
  all transferred remote data will appear in /usr/local/kdvs. This path is
  available in environment variable config\_file\_path\_dir.
- Evaluate user configuration file and preserve user variables.
- Configure logging facilities according to specification.

  If log file path is not specified, default log path
  $config\_file\_path\_dir/l1l2py\_experiment.log will be used.
- Initialize PPlusExecEnv instance according to specification; PPlus
  connection for storing files on remote disk is available as pplus\_connection
- Update environment variable storage with user variables.
- Resolve root output directory.

  If ‘:default:‘ is specified as value of result\_dir, then default
  output directory will be used; the default path has the format:
  $config\_file\_path\_dir/result\_\_<day>\_<month>\_<year>\_\_<hour>\_<min>\_<sec>/,
  when date and time components refer to directory creation time. Otherwise,
  specified path is used. This path is stored as root\_output\_path.
- Resolve database output directory.

  If -t TDIR command line option was used, TDIR will be used as directory
  where database files will be created and maintained during experiment
  execution. Otherwise, $root\_output\_path is used. This path is stored
  as tmpdb\_data\_dir.

  Note

  This directory must be writable.
- Emit all diagnostic messages collected earlier, before logger instance was
  created.
- Return environment instance.

### Actions¶

experiment is composed of the following actions, in add order:

| Name | Description |
| --- | --- |
| serialize\_cfg | Retrieve current environment variables and serialize them both with *PZP* protocol and in textual form; if variable value is not picklable (see pickle for details), a representation is serialized that allows the closest reconstruction of original object, as follows:   - for module – (module name, module file path) - for file handle – file name - for callable – (function name, function   source code if available) - for class – (class name, class source code   if available) |
| configure\_tablespaces | Initialize KDVS DB manager (KDVSDB) instance and create main experiment database |
| create\_tables | - Load all data files listed in configuration   file(s) into experiment database as raw data   tables, as follows: ANNO, GEDM,   HGNC, LABELS - Create derived tables, effectively performing   local data integration, in order:   term2probeset, probeset2gene |
| serialize\_misc\_objects | Create and serialize various utility mappings, both with *PZP* protocol and in textual form, in order: term2size, samples list, probesets list, labels mapping (if any), labels numerical vector (if any), probeset2geneid |
| build\_rls | Determine requested subsets of GO terms (dubbed ‘reading lists’), based on ANNO and GEDM data, as well as chosen strategy, and serialize them, both with *PZP* protocol and in textual form, in order: SIZE\_ABOVE, SIZE\_BELOW |
| generate\_submatrices | For every GO term in all reading lists, generate submatrix of original GEDM data that are associated with it, as well as all ranges of l1l2 parameters dependent on data, put them into meta-object, and serialize it with *PZP* protocol |
| process\_reading\_lists | For all recognized GO namespaces, process all reading lists of GO terms with chosen strategy; the strategy is customized for specific reading list and chosen statistical learning approach, and implemented as private function; see *Experiment methodology* for more details |
| transfer\_disk\_data | After all reading lists are processed, collect dispersed data from remote disk and copy to experiment master’s output directory |
| close\_tablespaces | Close all opened databases controlled by KDVS DB manager instance |

## postprocess¶

Implements the post-processing part of prototype framework for devising gene
signatures from microarray data while applying prior knowledge from Gene
Ontology. This part may be performed independently on results obtained from
experimental part, since it does not utilize parallel computational environment,
and no time-consuming statistical learning procedures are executed.

See *Methodology* for descriptive characteristics of the framework,
as well as for statistical concepts behind it.

It works as standalone command line application, with the following options:

|  |  |
| --- | --- |
| `-r RDIR` | perform postprocessing of experimental data in RDIR |
| `-c CFGFILE` | force reading configuration from CFGFILE |
| `-l LOGFILE` | direct log output to LOGFILE (with default log level INFO) |
| `--debug` | set log level to DEBUG |
| `--perform-FET` | perform Fisher Exact Test on selected nodes (Experimental) |
| `--FET-enrichment-pvalue` | |
|  | enrichment threshold for Fisher Exact Test (Experimental) |

Note

In -c option, postprocess accepts as CFGFILE only .cfg.py
configuration script (see *Configuring environment* for details)
and **not** *PZP*-serialized object. Because of this limitation,
after using experimental part of the framework, it is advised to preserve initial
configuration .cfg.py script, as well as *PZP*-serialized
initial configuration inside output of experimental part (unless re-configured,
it is saved as CFG there).

### Environment¶

postprocess uses LoggedExecEnv.

#### prepare\_env()¶

prepare\_env() function performs set of the following activities (when
referring to particular environment variable, $var\_name is the value of
variable identified by var\_name):

- Process command line arguments and set option values.
- Resolve input directory with the data to be post-processed.

  Check for presence of subdirectory <id>\_disk\_data inside given directory.
  If present, then use it as input directory. Otherwise, use given directory.
  This path is stored as kdvspp\_\_result\_dir.
- Create output directory where post-processing results will be written.

  The directory $kdvspp\_\_result\_dir/postprocessing\_results is created or reused.
  This path is stored as kdvspp\_\_final\_result\_dir.
- Configure logging facilities according to specification.

  If log file path is not specified, default log path
  $kdvspp\_\_final\_result\_dir/l1l2py\_experiment.log will be used.
- Initialize LoggedExecEnv instance according to specification.
- If -c CFGFILE option was used, store CFGFILE path as kdvspp\_\_cfg\_file.
- Return environment instance.

### Actions¶

postprocess is composed of the following actions, in add order; see
*Post-processing methodology* for more details:

| Name | Description |
| --- | --- |
| read\_cfg | Unless given with -c option, try to locate serialized configuration in input directory (by default, CFG). When successfully located, read configuration, either by deserializing with *PZP* protocol, or by execfile. Update environment variables with variables read from configuration |
| read\_probeset2geneid | Locate and read serialized object containing probeset2geneid mapping (see get\_probeset2geneid()) |
| read\_rls | Locate and read all subsets of GO terms (dubbed reading lists), and associate proper post-processing strategies (dubbed handler functions) for all of them; handlers are implemented as private functions |
| init\_global\_stat | Create meta-object that will hold all global statistics collected across the whole reading lists, as well as some meta-information as requested; this object is available as global\_stat |
| process\_rls | For all reading lists, execute proper handler functions that perform submatrix-focused post-processing part: collect all results from l1l2, calculate selected variables lists according to error and frequency thresholds, plot error surfaces, and calculate various statistical measurements |
| process\_stats | For all reading lists, execute proper handler functions that write result files with data collected across ranges of selected l1l2 parameters for more details |
| produce\_unified\_term\_lists\_for\_layer | In order to unify some results across different reading lists for some common values of l1l2 parameters, merge results from requested layers |
| collect\_global\_stat | Collect additional global statistics across all space of GO terms and l1l2 parameters, currently: histograms of selected and non-selected variables, and contingency tables for Fisher tests (**experimental**) |
| perform\_fisher\_exact\_test | If requested, perform Fisher exact test across all accepted results (**experimental**) |
| perform\_false\_discovery\_rate\_adjustment | If requested, perform false discovery rate adjustment on the results of Fisher exact test (**experimental**) |
| filter\_fisher\_exact\_test\_by\_thr | If requested, perform simple filtering of FDR results by specified threshold (**experimental**) |
| process\_go\_release | Read RDF-XML release of GO term hierarchy and term descriptions |
| write\_from\_global\_stat | Write any left-over information from global\_stat as readable text output, currently: unified term layers, and variable histograms |
| serialize\_global\_stat | For reference, serialize global\_stat meta-object, both with *PZP* protocol and as textual output |
| finish | Currently, emit ending log messages |

### utils¶

This module contains utility functions both for experiment and
postprocess applications. It must be available in the same directory
as those applications. The core API may be available either as local or installed
package kdvs.

## pzp\_dump¶

Dumps textual representation of Python object serialized to file with
*PZP* protocol. The textual representation is created using
pprint.

Usage:

```
> python pzp_dump.py /path/to/PZP_OBJ
Dumping pzp object /path/to/PZP_OBJ as /path/to/PZP_OBJ_T... done
>
```

### Table Of Contents

- Applications
  - experiment
    - Configuration file
    - Environment
      - Overview
      - prepare\_env()
    - Actions
  - postprocess
    - Environment
      - prepare\_env()
    - Actions
    - utils
  - pzp\_dump

### Quick search


Enter search terms or a module, class or function name.

### Navigation

- index
- modules |
- modules |
- next |
- previous |
- KDVS 0.0.1-alpha documentation »

© Copyright 2010-2012, Grzegorz Zycinski, Salvatore Masecchia, Annalisa Barla.
Created using Sphinx 1.1.2.
