# Supplementary material for: Knowledge Driven Variable Selection (KDVS) – a new approach to enrichment analysis of gene signatures obtained from high–throughput data
Source: Source Code Biol Med. 2013 Jan 9;8:2. doi: 10.1186/1751-0473-8-2 (PMC3605163; doi:10.1186/1751-0473-8-2)
Supplement: Additional file 1 — Source code of KDVS. Format: ZIP. It contains the Python source code, the documentation, and the internal data files. [file 1751-0473-8-2-S1.zip › KDVS/doc/_build/html/doc-gen/methodology.html]

Methodology — KDVS 0.0.1-alpha documentation


### Navigation

- index
- modules |
- modules |
- next |
- previous |
- KDVS 0.0.1-alpha documentation »

# Methodology¶

## Overview¶

The task of devising gene signatures from microarray data, while applying prior
knowledge to verify and further enrich them, may be solved in various ways,
regarding the method of utilizing prior knowledge.

The classic approach uses feature selection methods independently, to reduce size
of the problem, then applies additional information from prior knowledge
source(s) to verify selected variables in non-statistical terms:

Classic approach to solve the problem of devising gene signatures.

Any feature selection method may be used and any verification method may be applied.

This approach, while straightforward and elastic, requires careful choice of
feature selection method, based both on kind of data to process, as well as on
execution time, since every such method operates a priori on atomic
data sets (that can be very big).

The approach implemented in KDVS reverses the default order of using prior
knowledge, making it more elastic and more flexible in utilizing
different feature selection methods, as well as the usage of prior knowledge
itself:

KDVS approach to solve the problem of devising gene signatures

Any feature selection method may be used and any verification method may be applied.

The KDVS approach reduces the necessity of careful choosing the feature
selection technique, by specifying proper guided data rearrangement method.
In short, the original data set is partitioned into smaller subsets based on
specific information from prior knowledge source of choice. This step, both
data– and knowledge–dependent, can be refined by actual users of the data
to better reflect specific relations in prior knowledge.

For instance, expression data associated with specific gene can be coupled with
Gene Ontology terms connected with that gene (with this information coming from
Gene Ontology structural database). Therefore, original expression data set can
be partitioned into subsets associated with Gene Ontology terms, effectively
re–creating GO hierarchy of subsets. Besides producing much smaller datasets
for any feature selection method of choice, it allows further adjusting of even
more specific requirements.

For example, resulting subsets may be further partitioned according to their
physical size, to save computational time by using faster feature selection
technique on smaller ones. On the other hand, Gene Ontology terms themselves
may be associated with KEGG pathways of choice, introducing another hierarchical
level, if devising gene signatures is more focused on specific traits, e.g. in
various cancers, Alzheimer and Parkinson diseases etc, making the final solution
even more enriched.

By convention, the approach was decomposed into two non-overlapping parts:
experimental and post-process:

Decomposition of KDVS approach

## Experiment methodology¶

During experimental part, the following data sets are processed (in italic)
and the following computational techniques are used (in **bold**):

Conceptual schema of experimental part

### Raw data¶

#### Expression data¶

Expression data is produced by biological experiment that involves measurement
of the expression of genes, e.g. one performed on RNA microarrays, or directly
with next-generation sequencing (NGS) machines, or else.

For each biological sample, expression of many genes/sequences is measured
simultaneously, producing list of expression values for all genes/sequences
considered:

| <ID> | <SampleID> |
| --- | --- |
| <GeneID1> | 1.000001 |
| <GeneID2> | 3.999990 |
| <GeneID3> | 6.000300 |
| ... | ... |
| <CtlSeqID1> | 9.999999 |
| <CtlSeqID2> | 50.00001 |
| ... | ... |

After collecting all measurements from all samples, the expression values are
joined into one common matrix, dubbed gene expression data matrix (GEDM),
where each row or column contains expression data for specific gene or control
sequence across all samples considered. The matrix is then normalized using
well-established approaches available in BioConductor
R package.

KDVS Core API (*kdvs.core.GO.GEDM*) accepts normalized GEDM in DSV format, where
each row is associated with gene/sequence, hence the GEDM is expected to be
row-oriented:

| <ID> | <SampleID1> | <SampleID2> | <SampleID3> | <...> |
| --- | --- | --- | --- | --- |
| <GeneID1> | 1.00 | 2.00 | 3.00 | ... |
| <GeneID2> | 4.00 | 5.00 | 6.00 | ... |
| <GeneID3> | 7.00 | 8.00 | 9.00 | ... |
| ... | ... | ... | ... | ... |
| <CtlSeqID1> | 10.00 | 10.10 | 11.00 | ... |
| <CtlSeqID2> | 50.00 | 49.90 | 51.00 | ... |
| ... | ... | ... | ... | ... |

For details how actual DSV file with GEDM is parsed, see *Parsing metadata*.

#### Annotation data¶

KDVS can load and process annotation data, associated with expression data, in the
following formats:

- annotations provided by Affymetrix for their chips in standard format, see
  Affymetrix Annotation Files
  for more details
  - this annotation format is handled by ANNOFILE\_METADATA\_AFFY\_GPL
    metadata when loading
- annotations provided for platforms associated with experiments stored in GEO
  (Gene Expression Omnibus) (e.g.
  GPL96),
  in full mode (Download full table...)
  - this annotation format is handled by ANNOFILE\_METADATA\_GEO\_GPL
    metadata when loading

For details how actual files are parsed, see *Parsing metadata*.

#### Label information¶

Information regarding assignment of labels to biological samples is given in
CSV file with the following structure:

```
samples,labels
<sample_name1>,<label_value1>
<sample_name2>,<label_value2>
...
<sample_nameN>,<label_valueN>
```

If <label\_value> is equal to the value given in ignore\_label\_val parameter in
configuration file, then associated sample, and all its data, will be excluded
from all computations. Since expression data is row-oriented, excluding samples
equals to skipping certain columns in GEDM matrix.

### Guided data rearrangement¶

#### Overview¶

The expression data are associated with specific gene or control sequence.
Excluding control sequences and focusing on established gene names, we can
associate gene names with Gene Ontology terms:

```
Gene Ontology term  ->  Gene name   ->  (Expression values)
```

thus effectively build hierarchy of associated expression values in the form of
submatrices:

Identifier associations and submatrices hierarchy

For each GO term, rows of expression values associated with it (and, in order,
with gene names), are grouped together to form submatrices of expression values.

Each submatrix, associated with GO term, is stored as simple Python dictionary,
with the following keys:

- \_\_kdvs\_\_go\_namespace – Gene Ontology domain that contain associated GO term
- \_\_kdvs\_\_go\_term – short identifier of GO term (i.e. 0000001, **not** GO:0000001)
- \_\_kdvs\_\_samples – list of all samples, as loaded from GEDM (see
  get\_GEDM\_samples for more details)
- \_\_kdvs\_\_matrix – specific submatrix data, as dictionary of rows of
  expression values, keyed by probeset names (see get\_GEDM\_rows
  for more details)
- \_\_kdvs\_\_tau\_range – dynamically re-calculated range for l1l2 parameter tau
- \_\_kdvs\_\_mu\_range – dynamically re-calculated range for l1l2 parameter mu
- \_\_kdvs\_\_lambda\_range – stored range for l1l2 parameter lambda

Each submatrix object is self-contained, that is, all the data needed to perform
computational experiment on it and to post-process the results, are available
with it.

Finally, all submatrix objects are stored individually in *PZP* format.

#### Prior knowledge source¶

The guided rearrangement technique implemented in *experiment*
prototype application uses Gene Ontology RDF-XML release as prior knowledge
source. Currently, term hierarchy for all GO domains, and term names are
extracted from the source. The release is shipped with KDVS (by default, it is
located in $KDVS\_ROOT/data/GO\_release/go\_<reldate>-termdb.rdf-xml.gz, where
KDVS\_ROOT is the directory where KDVS is installed), but newer version may be
downloaded here
and replaced manually as needed.

### Feature selection/Regression¶

#### Overview¶

To select statistically meaningful features of the dataset that allow to build
good prediction model in supervised learning, one of the feature selection techniques is typically used,
to reduce size of the problem. However, in some cases, it is not needed to
perform computationally heavy feature selection for every single dataset
encountered; sometimes it is sufficient to perform simple estimation of
regression model, and accept/reject all features from dataset at once (dependent
on the outcome).

KDVS addresses this problem by dividing submatrices into groups: SIZE\_ABOVE and
SIZE\_BELOW (dubbed reading lists), based on their physical size (i.e.
number of rows of expression values), as follows:

- if the number of rows of submatrix is greater (>) than the value of parameter
  submatrices\_rows\_threshold in configuration file, the submatrix is put
  in SIZE\_ABOVE RL
- if the number of rows of submatrix is lesser or equal (<=) than the value of
  parameter submatrices\_rows\_threshold in configuration file, the submatrix
  is put in SIZE\_BELOW RL

For ‘smaller’ submatrices, there is no need to perform feature selection; a
simple regression will do:

- for bigger submatrices (SIZE\_ABOVE), l1l2 technique is used
- for smaller submatrices (SIZE\_BELOW), OLS technique is used

#### l1l2¶

l1l2 is a feature selection method that utilizes  regularization
with double optimization. It is used to select meaningful features in the dataset
according to presented outcome. KDVS uses l1l2 implementation from
L1L2Py package.

Note

This documentation will not deliberate on l1l2 details. See L1L2Py tutorial to get started,
and L1L2Py Algorithms
for more information. For theoretical aspects, consult references therein.

#### OLS¶

OLS (Ordinary Least Squares)
is a popular method for estimation of linear regression model. KDVS uses more
general implementation of Regularized Least Squares (RLS) from
L1L2Py package, with default
parameters, to perform OLS.

Note

See L1L2Py Regularization Algorithms
for more information.

#### Input¶

OLS operates on submatrices directly. l1l2, however, being double
optimization technique with two loops of cross validation, operates on splits,
that is, first the original submatrix is split on training and test subset at
random (see L1L2Py CV utilities
for technical details). The number of those ‘external’ splits is determined by
external\_k parameter specified in the configuration file (this parameter
is tunable). Later on, another cross validation is performed, and the number of
those ‘internal’ splits is determined by internal\_k parameter in the
configuration file (also tunable).

Note

At this moment, stratified splits are used, since it is not possible for KDVS
alone to determine balancing of statistical classes when doing classification.

To sum up:

- for l1l2, each ‘external’ split of submatrix is treated as atomic
  computational process, to be executed right away or in parallel
- for OLS, each model estimation, performed on the whole submatrix, is
  treated as atomic computational process, to be executed right away or in parallel

#### Output¶

In general, l1l2 returns the following for single ‘external’ split:

- mean CV errors for training and test ‘internal’ splits,
- calculated optimal values of tau and lambda parameters,
- list of models calculated for all mu values
- list of selected variables for each model

In general, OLS returns the following for single submatrix:

- prediction error
- predicted model

Note

For more detailed information, see
l1l2 specifics
and OLS specifics,
respectively.

### Selected features¶

#### Overview¶

When proper feature selection/regression technique is assigned to every submatrix,
and the atomic computational processes are determined, they are distributed
across local network of machines, controlled by PPlus environment:

Computation model of experimental part

#### l1l2¶

The atomic computational process for l1l2 performs as follows:

- extract numerical matrix from submatrix object
- creates ‘internal’ splits
- performs l1l2py.model\_selection
- collects results in output dictionary
- serializes output dictionary in PPlus shared disk space in *PZP* format

#### OLS¶

The atomic computational process for OLS performs as follows:

- extract numerical matrix from submatrix object
- performs l1l2py.algorithms.ridge\_regression
- collects results in output dictionary
- serializes output dictionary in PPlus shared disk space in *PZP* format

### Summary¶

After all atomic computational processes are finished, the experimental part
of KDVS framework concludes, and computational results are ready to be
post-processed.

## Post-processing methodology¶

The post-processing phase can be roughly divided in the following stages:

- collection of individual results, where data produced in experimental phase
  is reviewed, partial computational results are collected, and partial lists of
  selected variables are produced
- collection of global statistics, where various useful output data is produced,
  such as variable histograms, unified lists of selected variables, etc
- performing additional activities as needed, based on collected individual and
  global results/statistics

Overview of experimental data before post-processing part

Overview of post-processing data produced

Overview of post-processing data produced for l1l2 results

Overview of post-processing data produced for OLS results

### Individual results¶

Processing of individual results obtained for each submatrix, regardless the
statistical procedure used, comprises of the following steps:

- data review
- error reconstruction
- error threshold verification
- error plotting (if applicable)
- frequency verification (if applicable)

Note

Not all submatrices will pass the error threshold verification step.

First, *postprocess* reads configuration file produced
during experimental phase (either found CFG or the one provided), and
reconstructs reading lists of submatrices.

Then, depending on particular reading list, it performs the following activities:

- for each submatrix from SIZE\_ABOVE:

  - read submatrix object and reconstruct numerical matrix
  - based on external\_k configuration parameter, check if all external
    splits were distributed and if all partial results were retrieved; if not,
    discard the submatrix from further post-processing (**data review**)
  - read all partial results from splits (**error reconstruction**) and
    calculate the following:

    - average error for internal l1l2 splits, for training and test part
    - mean of prediction error, for training and test part
    - standard deviation of prediction error, for training and test part
    - median of prediction error, for test part
  - for all values of l1l2 mu parameters for that submatrix, compare
    mean of prediction error for test part (avg\_err\_ts) with given threshold
    (specified in threshold\_error configuration parameter):

    - if avg\_err\_ts < threshold\_error for particular mu value,
      record that value as valid
    - if avg\_err\_ts >= threshold\_error for particular mu value,
      reject that value

    if at least one mu value was recorded as valid, proceed; otherwise,
    discard the submatrix from further post-processing (**error threshold
    verification**)

    the avg\_err\_ts values for valid mus are stored to be later provided
    as error estimate in global results
  - if submatrix has passed error threshold verification step, plot the following
    error surfaces:

    - for average error for internal l1l2 splits (training and test part)
    - for prediction error (training and test part)
  - for every valid mu value recorded, calculate frequency scores for
    selected features or variables, that is: count the number of appearances
    of particular variable in all nested variable lists produced by l1l2,
    present it as percentage (freq\_perc), and compare it with given threshold
    (specified in threshold\_frequency configuration parameter):

    - if freq\_perc > threshold\_frequency for particular variable,
      mark that variable as being properly selected
    - if freq\_perc <= threshold\_frequency for particular variable,
      mark that variable as being not selected

    only selected variables (i.e. that appear frequent enough) are reported
    as proper output (**frequency verification**); however, some global
    statistics are still collected also for non-selected variables
- for each submatrix from SIZE\_BELOW:

  - read submatrix object and reconstruct numerical matrix
  - check if single partial result was retrieved; if not, discard the submatrix
    from further post-processing (**data review**)
  - read partial result (**error reconstruction**)
  - compare single prediction error value (err) with given threshold
    (specified in threshold\_error configuration parameter):

    - if err < threshold\_error, accept the submatrix and proceed
    - if err >= threshold\_error, reject the submatrix and discard it
      from further post-processing (**error threshold verification**)

    the err value is stored to be later provided as error estimate in
    global results
  - if submatrix was accepted, all its features or variables are reported
    as being properly selected; otherwise, the variables are reported as
    not selected

#### Individual output¶

- for each submatrix from SIZE\_ABOVE, separated folder is created in
  postprocessing\_results root subdirectory, and the following individual files
  are written there:

  - error surface plot of average error for internal l1l2 splits, both
    for training and test part, versus l1l2 lambda and tau parameters
  - box plot of prediction error, for training part, versus l1l2 mu
    parameter
  - box plot of prediction error, for test part, versus l1l2 mu
    parameter
  - for each valid mu value, the partial list of selected variables is
    produced, in the following format:

    ```
    <mu value> <number of variables(N)>
    <probeset_id1> <gene_name(s)> <entrez_gene_id(s)> <genbank_acc_id> <absolute_frequency_in_%>
    <probeset_id2> <gene_name(s)> <entrez_gene_id(s)> <genbank_acc_id> <absolute_frequency_in_%>
    ...
    <probeset_idN> <gene_name(s)> <entrez_gene_id(s)> <genbank_acc_id> <absolute_frequency_in_%>
    ```

    For instance:

    ```
    0.023315    12
    223055_s_at XPO5    57510   AF271159    100
    201475_x_at MARS    4141    NM_004990   100
    215208_x_at RPL35A  6165    AK021571    100
    200079_s_at KARS    3735    AF285758    100
    212160_at   XPOT    11260   AI984005    100
    204283_at   FARS2   10667   NM_006567   75
    223076_s_at NSUN2   54888   BC001041    75
    223015_at   EIF2A   83939   AF212241    50
    201139_s_at SSB 6741    NM_003142   50
    202541_at           BF589679    25
    238760_at   YARS    8565    AW452122    25
    201000_at   AARS    16  NM_001605   25
    ```

    Note

    Entrez Gene IDs and gene names may **not** be available; in that case,
    GenBank Accession ID can be used to identify the sequence.
- for SIZE\_BELOW, a folder named size\_below is created in
  postprocessing\_results root subdirectory, and for each submatrix from
  SIZE\_BELOW, the following files are written there:

  - the partial list of selected variables in the following format:

    ```
    <probeset_id1> <gene_name(s)> <entrez_gene_id(s)> <genbank_acc_id>
    <probeset_id2> <gene_name(s)> <entrez_gene_id(s)> <genbank_acc_id>
    ...
    <probeset_idN> <gene_name(s)> <entrez_gene_id(s)> <genbank_acc_id>
    ```

    For instance:

    ```
    203719_at   ERCC1   2067    NM_001983
    203720_s_at ERCC1   2067    NM_001983
    205395_s_at MRE11A  4361    NM_005590
    208393_s_at RAD50   10111   NM_005732
    209349_at   RAD50   10111   U63139
    211334_at   MRE11A  4361    BC005241
    228131_at   ERCC1   2067    BG111047
    242456_at   MRE11A  4361    AA931565
    ```

    Note

    Entrez Gene IDs and gene names may **not** be available; in that case,
    GenBank Accession ID can be used to identify the sequence.

### Global Statistics¶

Global statistics are collected over all submatrices and all partial results
collected.

By convention, *postprocess* uses meta dictionaries
to collect information within specified range:

- <RL\_id>\_stats – to collect reading list-wide information; in current
  prototype, SIZE\_ABOVE\_stats and SIZE\_BELOW\_stats objects are used
- global\_stat – to collect global-wide information that crosses reading
  list domain

Within the reading list range, the following information is collected:

- reading list itself, in the form of submatrices IDs, for reference
- global list of submatrices that pass error threshold verification
- global list of submatrices that do not pass error threshold verification
- number of degrees of freedom (currently, number of mu values for
  l1l2 and 1 for OLS) for producing unified variables lists

> Note
>
> Partial lists of selected variables are often collected regarding the value
> of some parameter; in the KDVS prototype, for example, for l1l2 partial
> lists will differ for different mu values. To facilitate combining
> the content of partial lists across different reading lists into unified
> lists, the idea of degrees of freedom (DOF) was introduced, that is:
>
> - if the content of partial lists depends on range of values of some
>   parameters (and in order, separate lists are produced for different
>   parameter values), then every parameter value is considered as one
>   degree of freedom, and is indexed starting from 0
> - if partial list does not depend on any parameter, then the single
>   degree of freedom defaults to 0
>
>   For example, if reading list RL1 depends on the following range of
>   parameter values:
>
>   ```
>   0.1, 0.001, 0.000001
>   ```
>
>   and the second one (RL2) does not depend on anything (i.e. partial list
>   is constant), the following holds:
>
>   > - the degrees of freedom for RL1 are (0.1, 0.001, 0.000001),
>   >   indexed as (0, 1, 2); the DOF number is 3
>   > - the degrees of freedom for RL2 are (0), indexed as (0); the DOF
>   >   number is 1

- for each degree of freedom, the list of submatrices that pass error
  threshold verification (for l1l2, for each mu value, for OLS,
  for value 0)
- for SIZE\_ABOVE, some additional data regarding errors is collected for
  every submatrix separately to facilitate producing global output data (see
  Global Output for details)

The following global statistics are collected across all experimental data; some
information may be redundant:

- for every submatrix, and every degree of freedom:
  - total number of variables associated with submatrix
  - number of selected variables
  - list of selected variables IDs
  - list of not selected variables IDs
- for each requested combination of degrees of freedom across different reading
  lists, the unified variables lists

> Note
>
> For instance, for RL1 the following partial lists of selected variables were
> produced:
>
> ```
> (V1, V2, V3) for DOF 0.1
> (V4, V5, V6) for DOF 0.001
> (V7, V8, V9) for DOF 0.000001
> ```
>
> And for RL2 the following single list was produced:
>
> ```
> (W1, W2, W3) for DOF 0
> ```
>
> The following unified variable lists may be produced:
>
> ```
> (V1, V2, V3, W1, W2, W3) for RL1 DOF 0 and RL2 DOF 0
> (V4, V5, V6, W1, W2, W3) for RL1 DOF 1 and RL2 DOF 0
> (V7, V8, V9, W1, W2, W3) for RL1 DOF 2 and RL2 DOF 0
> ```
>
> Each DOF merge is done upon explicit request.

- set of variables selected at least once in any submatrix
- set of variables that were never selected anywhere
- for each degree of freedom, set of variables selected at least once in
  submatrices that passed error threshold verification
- for each degree of freedom, set of variables never selected in
  submatrices that passed error threshold verification
- for each degree of freedom, a full histogram of all selected values across
  submatrices that passed error threshold verification (number of selected
  variable appearances across submatrices is counted)
- for each degree of freedom, a full histogram of all not selected values across
  submatrices that passed error threshold verification (number of not selected
  variable appearances across submatrices is counted)
- (**experimental**) 2x2 contingency tables for all single submatrices,
  calculated as follows:

  |  |  |
  | --- | --- |
  | Number of selected variables for particular submatrix | Number of not selected variables for particular submatrix |
  | Number of variables selected outside particular matrix | Number of variables not selected outside particular matrix |

  Contingency tables are calculated only if performing FET was requested with
  --perform-FET command line option for postprocess application.

  See Fisher Exact Test for more details.

#### Global Output¶

Currently, the following files are produced:

- for each l1l2 mu value, the SIZE\_ABOVE\_stats\_mu[X] CSV file with
  header, that contains the following columns:

  - full GO term ID (i.e. GO:0000001)
  - mu value index
  - mean of test error
  - standard deviation of test error
  - mean of prediction error
  - standard deviation of prediction error
  - median of test error
  - total number of variables associated with the submatrix
  - total number of selected variables associated with the submatrix

  For instance:

  ```
  GO term ID,Mu,Mean TS,Std TS,Mean TR,Std TR,Med TS,Tot vars,Sel vars
  GO:0005515,0,0.0595927312902,0.0237055815607,0.0,0.0,0.0624713039486,15035,230
  GO:0046872,0,0.045928030303,0.0265278311133,0.00520912987994,0.00521768678784,0.0606060606061,6596,98
  GO:0008270,0,0.0693583562902,0.033817940487,0.00781221192909,0.00851309206943,0.0625,5250,87
  GO:0003677,0,0.044951467803,0.0161935917425,0.0104182597599,0.00708073456502,0.047881155303,4895,62
  GO:0000166,0,0.0478739812902,0.033404962323,0.013073940168,0.0173600747312,0.049834280303,4479,65
  ```

  The terms are associated with submatrices, and are in descending order regarding
  size (starting from the biggest submatrix down to the smallest one; when two
  or more submatrices have the same size, they are written in random).
- for each DOF merge request, the unified term list unified\_term\_list\_[RL1]\_[DOF\_index\_1]\_[RL2]\_[DOF\_index\_2]
  TSV file with header, that contains the following columns:

  - full GO term ID (i.e. GO:0000001)
  - GO term name
  - total number of variables associated with the submatrix
  - total number of selected variables associated with the submatrix
  - an error estimate obtained for the submatrix

  For instance:

  ```
  GO term ID  GO term name    Tot vars    Sel vars    Error estimate
  GO:0005515  protein binding 5988    43  0.286454475309
  GO:0046872  metal ion binding   1962    258 0.284691358025
  GO:0000166  nucleotide binding  1744    442 0.269992283951
  GO:0003677  DNA binding 1727    393 0.281219135802
  GO:0005524  ATP binding 1362    137 0.251674382716
  GO:0004872  receptor activity   1312    171 0.255177469136
  ```

  The terms are associated with submatrices, and are in descending order regarding
  size (starting from the biggest submatrix down to the smallest one; when two
  or more submatrices have the same size, they are written in random).
- depending on the reading list and degrees of freedom, the histograms of
  selected/not selected variables:

  - for SIZE\_ABOVE and mu, selected\_vars\_hist\_SIZE\_ABOVE\_[X] and
    not\_selected\_vars\_hist\_SIZE\_ABOVE\_[X] files (in total 2\*K files,
    where K is the number of different mu values)
  - for SIZE\_BELOW, single selected\_vars\_hist\_SIZE\_BELOW\_0 file (all
    variables are selected once submatrices from this reading list are passing
    error threshold verification, therefore histogram of not selected variables
    cannot be compiled for this RL)

  All histogram files have similar format:

  ```
  [Selected|Not selected] at least once: [N]    Nodes passing TS error: [P]   Nodes in <GO_namespace(s)>: [R]
  <probeset_id1> <gene_name(s)> <histogram_counter>
  ...
  <probeset_idN> <gene_name(s)> <histogram_counter>
  ```

  For instance, for histogram of selected variables:

  ```
  Selected at least once: 2636    Nodes passing TS error: 104   Nodes in MF:8663
  208860_s_at ATRX    6
  208859_s_at ATRX    6
  213093_at   PRKCA   6
  201945_at   FURIN   6
  201183_s_at CHD4    6
  ```

  And for histogram of not selected variables:

  ```
  Not selected at least once: 8881    Nodes passing TS error: 104  Nodes in MF:8663
  208195_at   TTN 8
  213468_at   ERCC2   8
  31861_at    IGHMBP2 7
  205065_at   ENPP1   7
  215980_s_at IGHMBP2 7
  ```

  The variables are ordered in decreasing order of histogram counters.

  Nodes passing TS error corresponds to the number of submatrices passing
  error threshold verification for this mu value.

  Nodes in <GO\_namespace(s)> corresponds to the total number of GO terms in
  specified GO domains,
  as read from current GO release (see Prior knowledge source for technical
  details); GO domains to process are specified in go\_namespaces parameter in
  configuration file

### Additional activities (**Experimental**)¶

Currently, *postprocess* implements additional level of
statistical verification of results, in the form of Fisher Exact Test
(FET).

Note

This functionality is treated as **unstable** and is **disabled** by default.
To enable it, specify --perform-FET command line option when
starting postprocess application. R and
rpy2 must be installed to use it.

For performing Fisher Exact Test and related statistical procedures, KDVS
uses R stats
package. It is accessed through KDVS core API module rint,
a simple wrapper over rpy2 Python to R interface. See
rpy2 documentation
for more details.

The reason for using R stats was its robustness in handling large
factorials.

#### Fisher Exact Test¶

Based on 2x2 contingency tables calculated for all submatrices, the test statistics
(p-value) is calculated as follows:

> |  |  |  |  |
> | --- | --- | --- | --- |
> |  |  |  | Totals |
> |  | Number of selected variables for particular submatrix (**a**) | Number of not selected variables for particular submatrix (**b**) | a + b |
> |  | Number of variables selected outside particular submatrix (**c**) | Number of variables not selected outside particular submatrix (**d**) | c + d |
> | Totals | a + c | b + d | a + b + c + d = n |

KDVS uses robust fisher.test
implementation of Fisher Exact Test to obtain p-value (p-value) and odds ratio
(estimate), respectively. One p-value and one odds ratio value are assigned
to each submatrix.

#### Multiple testing correction¶

Since many singular FET tests are performed simultaneously, and statistics in
contingency tables depend on each other, there is a need for correcting default
p-values received from fisher.test.
KDVS uses p.adjust
to do so, with fdr as default adjustment method.

#### P-value threshold filtering¶

Adjusted p-values are filtered regarding p-value threshold, specified as
threshold\_enrichment\_pvalue parameter in configuration file, or directly
(currently set as 0.05 in *postprocess*), as follows:

- if adjusted p-value < threshold\_enrichment\_pvalue, the submatrix passes FET
- if adjusted p-value >= threshold\_enrichment\_pvalue, the submatrix does not
  pass FET

#### Output¶

At this moment, the results are not reported in the global output. However, they
are still available in global\_stat meta-dictionary.

### Table Of Contents

- Methodology
  - Overview
  - Experiment methodology
    - Raw data
      - Expression data
      - Annotation data
      - Label information
    - Guided data rearrangement
      - Overview
      - Prior knowledge source
    - Feature selection/Regression
      - Overview
      - l1l2
      - OLS
      - Input
      - Output
    - Selected features
      - Overview
      - l1l2
      - OLS
    - Summary
  - Post-processing methodology
    - Individual results
      - Individual output
    - Global Statistics
      - Global Output
    - Additional activities (**Experimental**)
      - Fisher Exact Test
      - Multiple testing correction
      - P-value threshold filtering
      - Output

### Quick search


Enter search terms or a module, class or function name.

### Navigation

- index
- modules |
- modules |
- next |
- previous |
- KDVS 0.0.1-alpha documentation »

© Copyright 2010-2012, Grzegorz Zycinski, Salvatore Masecchia, Annalisa Barla.
Created using Sphinx 1.1.2.
