# Supplementary material for: Knowledge Driven Variable Selection (KDVS) – a new approach to enrichment analysis of gene signatures obtained from high–throughput data
Source: Source Code Biol Med. 2013 Jan 9;8:2. doi: 10.1186/1751-0473-8-2 (PMC3605163; doi:10.1186/1751-0473-8-2)
Supplement: Additional file 1 — Source code of KDVS. Format: ZIP. It contains the Python source code, the documentation, and the internal data files. [file 1751-0473-8-2-S1.zip › KDVS/doc/_build/html/doc-gen/overview.html]

Overview — KDVS 0.0.1-alpha documentation


### Navigation

- index
- modules |
- modules |
- next |
- previous |
- KDVS 0.0.1-alpha documentation »

# Overview¶

## Idea¶

The main idea behind KDVS is data and knowledge integration.

Modern biology tries to understand dense web of molecular interactions to uncover
underlying structure and mechanisms that describe biological organisms. This can
be done just for the sake of pure knowledge, or for more practical purpose,
such as searching for a cure that treats particular disease.

To do so, life science researchers need two important sources of information:
data and knowledge. Data typically comes from measurement of various kinds of
biological activities, and is available in numerical form. Knowledge typically
comes directly from researchers that try to understand biological mechanisms
and to codify the resulting processes in various forms.

The form of both sources of information is usually distinct. Numerical data is
non-readable directly, but provides wealth of informative relations that may be
hidden to human researchers. Knowledge base, while readable directly by any
researcher, may be difficult to adapt for numerical computational processes,
typically applied to data. That makes the open problem on its own: how to
integrate data and knowledge effectively? See, for example,
this editorial
for an introduction to the topic.

### High-throughput platforms¶

KDVS operates on data produced by high-throughput platforms (HTP). HTP is a
workflow system utilized in modern life sciences, composed of hardware and
software, that quantifies many instances of biological/physical phenomena
simultaneously. For example, the amount of RNA associated with many genes can
be measured simultaneously on array-like plates, or many ions may be identified
simultaneously during mass spectrometry of the peptide, or many short reads may
be generated and assembled into contigs during next-gen sequencing.

Typical run of HTP produces very big data streams (terabyte-level is not a rare
case) that need to be processed to reveal properties useful for understanding the
underlying phenomena. This may be done immediately after data production phase,
using tools provided by HTP manufacturer. However, it is possible to use other
tools, if the data stream itself is available and data stream format is known.

Many manufacturers use proprietary data formats for data streams produced by
HTPs. However, it is very often the case that:

- manufacturer publishes data format specification
- manufacturer offers public API to handle its data format
- manufacturer enables data to be exported into portable formats, like DSV
  (delimiter separated values)

The overall stream of data produced by HTP can be roughly divided in two parts:
numerical data and annotation metadata. Numerical data are direct results of
simultaneous measures of quantities. Annotation data provides additional
information related to those results, such as: what quantities those particular
numbers measure, what is the meaning of this measure, what are the environmental
features associated with those measures, what are the external concepts
associated with those measures etc.

Both numerical and annotation data are available in many binary or textual
formats, DSV being the most widespread textual format.

## Input Data¶

### Delimiter Separated Values¶

DSV stores two-dimensional arrays of data, or two-dimensional data slices in
case of multi-dimensional data, in rows of plain text elements, separating
them with special delimiter characters. For example:

```
0.00001,0.00002,"Example data string",'33333'
333,"Another data string",+++,0.55,111,"Tricky, but possible"
```

Here, the delimiter character is comma ”,”, and the data are stored
as CSV (comma-separated values). Among other standard delimiter characters are
tabulator “\t” (TSV, tabulator-separated values, accordingly), colon
”.” and pipe “|”.

Many spreadsheet and database applications allow writing data in DSV portable
formats. Since the most common “dialect” of DSV is CSV, it will be often
referred as such.

Very often, the CSV data file is coming up with a “header line”, containing
names for the “columns” of values, and “row names”, containing the name for
every row. For example:

```
"Row ID","Column1","Column2","Column3"
"Row1",1.00,2.00,3.00
"Row2",4.00,5.00,6.00
```

In this case, Column1 represents the set of values 1.00, 4.00, Column2
represents the set of values 2.00, 5.00, etc; Row1 represents the set of
values 1.00, 2.00, 3.00 etc.

### Numerical data¶

Numerical data, typically being huge, may be stored and transmitted in some
compact binary format by correspondent HTP technology, but in most cases it will
be available also in DSV format, as a simple series of numerical values:

```
"Row ID","Column1","Column2","Column3"
"Row1",1.00,2.00,3.00
"Row2",4.00,5.00,6.00
```

Currently, KDVS processes numerical data in the form of properly formed DSV files.
The format of the data is described with *Parsing metadata*.

Note

It is often the case that numerical CSV data file comes as output from R
write.table family of functions. With the default set of options for those
functions, the header line, if present, contains one column less. For example:

```
"Column1","Column2","Column3"
"Row1",1.00,2.00,3.00
"Row2",4.00,5.00,6.00
```

Such CSV file is not conforming to standard CSV formatting rules. Therefore,
to be processed by KDVS, it must contain full header line, even if there is
no name for the first column. Here, the first column name is empty string:

```
,"Column1","Column2","Column3"
"Row1",1.00,2.00,3.00
"Row2",4.00,5.00,6.00
```

R documentation regarding writing CSV files:

- R Data Import/Export
- write.table

### Annotation metadata¶

“Raw” stream of numerical data, however, is not very useful. Additional
information about this data must be provided, such as: what quantities those
particular numbers measure, what is the meaning of this measure, what are the
environmental features associated with those measures, what are the external
concepts associated with those measures etc. This information is provided
in the form of annotation metadata.

Unlike simple numerical measures, annotation metadata may contain much richer
information, such as: arbitrary text strings of any format, date and time strings,
record-like substructures etc.

Typically, annotation metadata comes in the form of DSV files, so all the remarks
mentioned before apply here as well. However, since the information is much more
complicated, it takes considerable amount of time to parse properly and to
extract all relevant information.

Below is the complete example of annotation metadata in TSV format that
accompanies gene expression measurements performed on Affymetrix microarray
chip; presented are header line and two data
lines:

```
ID  GB_ACC  SPOT_ID Species Scientific Name Annotation Date Sequence Type   Sequence Source Target Description  Representative Public ID    Gene Title  Gene Symbol ENTREZ_GENE_ID  RefSeq Transcript ID    Gene Ontology Biological Process    Gene Ontology Cellular Component    Gene Ontology Molecular Function
1007_s_at   U48705      Homo sapiens    Mar 11, 2009    Exemplar sequence   Affymetrix Proprietary Database U48705 /FEATURE=mRNA /DEFINITION=HSU48705 Human receptor tyrosine kinase DDR gene, complete cds U48705  discoidin domain receptor tyrosine kinase 1 DDR1    780 NM_001954 /// NM_013993 /// NM_013994   0006468 // protein amino acid phosphorylation // inferred from electronic annotation /// 0007155 // cell adhesion // traceable author statement /// 0007155 // cell adhesion // inferred from electronic annotation /// 0007169 // transmembrane receptor protein tyrosine kinase signaling pathway // inferred from electronic annotation  0005887 // integral to plasma membrane // traceable author statement /// 0016020 // membrane // inferred from electronic annotation /// 0016021 // integral to membrane // inferred from electronic annotation  0000166 // nucleotide binding // inferred from electronic annotation /// 0004672 // protein kinase activity // inferred from electronic annotation /// 0004713 // protein tyrosine kinase activity // inferred from electronic annotation /// 0004714 // transmembrane receptor protein tyrosine kinase activity // traceable author statement /// 0004714 // transmembrane receptor protein tyrosine kinase activity // inferred from electronic annotation /// 0004872 // receptor activity // inferred from electronic annotation /// 0005515 // protein binding // inferred from physical interaction /// 0005524 // ATP binding // inferred from electronic annotation /// 0016301 // kinase activity // inferred from electronic annotation /// 0016740 // transferase activity // inferred from electronic annotation
1053_at M87338      Homo sapiens    Mar 11, 2009    Exemplar sequence   GenBank M87338 /FEATURE= /DEFINITION=HUMA1SBU Human replication factor C, 40-kDa subunit (A1) mRNA, complete cds    M87338  replication factor C (activator 1) 2, 40kDa RFC2    5982    NM_002914 /// NM_181471 0006260 // DNA replication // not recorded /// 0006260 // DNA replication // inferred from electronic annotation /// 0006297 // nucleotide-excision repair, DNA gap filling // not recorded 0005634 // nucleus // inferred from electronic annotation /// 0005654 // nucleoplasm // not recorded /// 0005663 // DNA replication factor C complex // inferred from direct assay /// 0005663 // DNA replication factor C complex // inferred from electronic annotation   0000166 // nucleotide binding // inferred from electronic annotation /// 0003677 // DNA binding // inferred from electronic annotation /// 0003689 // DNA clamp loader activity // inferred from electronic annotation /// 0005515 // protein binding // inferred from physical interaction /// 0005524 // ATP binding // traceable author statement /// 0005524 // ATP binding // inferred from electronic annotation /// 0017111 // nucleoside-triphosphatase activity // inferred from electronic annotation
```

Currently, KDVS processes annotation metadata in the form of properly formed DSV
files, as well as in some few more exotic formats, when proper parsing methodology
is available. The format of the data is described with *Parsing metadata*.

### Table Of Contents

- Overview
  - Idea
    - High-throughput platforms
  - Input Data
    - Delimiter Separated Values
    - Numerical data
    - Annotation metadata

### Quick search


Enter search terms or a module, class or function name.

### Navigation

- index
- modules |
- modules |
- next |
- previous |
- KDVS 0.0.1-alpha documentation »

© Copyright 2010-2012, Grzegorz Zycinski, Salvatore Masecchia, Annalisa Barla.
Created using Sphinx 1.1.2.
