# Supplementary material for: Knowledge Driven Variable Selection (KDVS) – a new approach to enrichment analysis of gene signatures obtained from high–throughput data
Source: Source Code Biol Med. 2013 Jan 9;8:2. doi: 10.1186/1751-0473-8-2 (PMC3605163; doi:10.1186/1751-0473-8-2)
Supplement: Additional file 1 — Source code of KDVS. Format: ZIP. It contains the Python source code, the documentation, and the internal data files. [file 1751-0473-8-2-S1.zip › KDVS/doc/_build/html/genindex.html]

Index — KDVS 0.0.1-alpha documentation


### Navigation

- index
- modules |
- modules |
- KDVS 0.0.1-alpha documentation »

# Index

**A**
| **C**
| **D**
| **E**
| **F**
| **G**
| **I**
| **K**
| **L**
| **N**
| **P**
| **Q**
| **R**
| **S**
| **T**
| **U**
| **V**

## A

|  |  |
| --- | --- |
| add\_action() (kdvs.core.execenv.execenv.ExecEnv method) | add\_var() (kdvs.core.execenv.execenv.ExecEnv method) |

## C

|  |  |
| --- | --- |
| clear\_actions() (kdvs.core.execenv.execenv.ExecEnv method)  close() (kdvs.core.db.KDVSDB method)  collect\_immediate\_children() (in module kdvs.core.GO.GOTermTreeManip)  collect\_subtree\_terms() (in module kdvs.core.GO.GOTermTreeManip)  collect\_subtree\_terms\_with\_depth() (in module kdvs.core.GO.GOTermTreeManip) | collect\_term\_parents() (in module kdvs.core.GO.GOTermTreeManip)  copy\_table() (kdvs.core.db.KDVSDB method)  create\_csv\_table\_st\_from\_schema() (in module kdvs.core.util)  create\_probeset2gene() (in module kdvs.core.GO.HGNC)  create\_term2probeset() (in module kdvs.core.GO.annotation) |

## D

|  |  |
| --- | --- |
| db\_provider (class in kdvs.core.provider)  del\_var() (kdvs.core.execenv.execenv.ExecEnv method) | deserialize\_pzp\_from\_filekey() (kdvs.core.execenv.pplus\_env.PPlusExecEnv method) |

## E

|  |  |
| --- | --- |
| evaluate\_cfg\_file() (in module kdvs.core.config)  ExecEnv (class in kdvs.core.execenv.execenv) | execute() (kdvs.core.execenv.execenv.ExecEnv method)  execute\_all() (kdvs.core.execenv.execenv.LoggedExecEnv method) |

## F

|  |  |
| --- | --- |
| file\_provider() (in module kdvs.core.provider)  find\_str\_in\_seq() (in module kdvs.core.util)  format\_action\_spec() (kdvs.core.execenv.execenv.ExecEnv method) | fpBzip2File (class in kdvs.core.provider)  fpGzipFile (class in kdvs.core.provider) |

## G

|  |  |
| --- | --- |
| get\_default\_cfg\_file\_path() (in module kdvs.core.config)  get\_default\_GO\_termdb\_release() (in module kdvs.core.config)  get\_default\_internal\_data\_root\_path() (in module kdvs.core.config)  get\_default\_R\_data\_root\_path() (in module kdvs.core.config)  get\_default\_vis\_data\_root\_path() (in module kdvs.core.config)  get\_GEDM\_probesets() (in module kdvs.core.GO.GEDM)  get\_GEDM\_rows() (in module kdvs.core.GO.GEDM)  get\_GEDM\_samples() (in module kdvs.core.GO.GEDM)  get\_geneid() (in module kdvs.core.GO.HGNC) | get\_labels() (in module kdvs.core.GO.subm)  get\_ns\_terms() (in module kdvs.core.GO.annotation)  get\_probeset2geneid() (in module kdvs.core.GO.HGNC)  get\_probesets() (in module kdvs.core.GO.annotation)  get\_subm\_for\_term() (in module kdvs.core.GO.subm)  get\_term2probeset() (in module kdvs.core.GO.annotation)  get\_term2size() (in module kdvs.core.GO.annotation)  get\_tmpdb() (kdvs.core.db.KDVSDB method)  get\_tmpdb\_loc() (kdvs.core.db.KDVSDB method) |

## I

|  |
| --- |
| is\_metadata() (in module kdvs.core.util) |

## K

|  |  |
| --- | --- |
| kdvs.core.config (module)  kdvs.core.db (module)  kdvs.core.error (module)  kdvs.core.execenv (module)  kdvs.core.execenv.execenv (module)  kdvs.core.execenv.pplus\_env (module)  kdvs.core.GO.annotation (module)  kdvs.core.GO.GEDM (module)  kdvs.core.GO.GOTermTree (module)  kdvs.core.GO.GOTermTreeManip (module) | kdvs.core.GO.HGNC (module)  kdvs.core.GO.subm (module)  kdvs.core.metadata (module)  kdvs.core.provider (module)  kdvs.core.rint (module)  kdvs.core.util (module)  KDVSDB (class in kdvs.core.db)  KDVSError  KDVSMetadata (class in kdvs.core.provider)  KDVSWarning |

## L

|  |  |
| --- | --- |
| load\_csv() (kdvs.core.db.KDVSDB method) | LoggedExecEnv (class in kdvs.core.execenv.execenv) |

## N

|  |
| --- |
| NullOutputStream (class in kdvs.core.util) |

## P

|  |  |
| --- | --- |
| parse\_go\_rdf\_xml() (in module kdvs.core.GO.GOTermTree)  PPlusExecEnv (class in kdvs.core.execenv.pplus\_env)  pprint\_to\_filekey() (kdvs.core.execenv.pplus\_env.PPlusExecEnv method)  probeset2gene\_schema (in module kdvs.core.db)  probeset2gene\_table (in module kdvs.core.db) | Python Enhancement Proposals  PEP 249  pzp\_deserialize() (in module kdvs.core.util)  pzp\_deserialize\_obj() (in module kdvs.core.util)  pzp\_serialize() (in module kdvs.core.util)  pzp\_serialize\_obj() (in module kdvs.core.util) |

## Q

|  |
| --- |
| quote() (in module kdvs.core.util) |

## R

|  |  |
| --- | --- |
| R() (in module kdvs.core.rint)  Rcall() (in module kdvs.core.rint) | Rimport() (in module kdvs.core.rint)  Robj() (in module kdvs.core.rint) |

## S

|  |  |
| --- | --- |
| serialize\_pzp\_to\_filekey() (kdvs.core.execenv.pplus\_env.PPlusExecEnv method)  serialize\_txt\_to\_filekey() (kdvs.core.execenv.pplus\_env.PPlusExecEnv method)  skip\_comment\_reader() (in module kdvs.core.util)  sniff\_dsv() (in module kdvs.core.util) | sqlite3\_db\_provider (class in kdvs.core.provider)  sqlite3\_provider\_cfg (in module kdvs.core.provider)  subtree() (in module kdvs.core.GO.GOTermTreeManip) |

## T

|  |  |
| --- | --- |
| term2probeset\_schema (in module kdvs.core.db) | term2probeset\_table (in module kdvs.core.db) |

## U

|  |
| --- |
| update\_vars() (kdvs.core.execenv.execenv.ExecEnv method) |

## V

|  |  |
| --- | --- |
| var() (kdvs.core.execenv.execenv.ExecEnv method) | varkeys() (kdvs.core.execenv.execenv.ExecEnv method) |

### Quick search


Enter search terms or a module, class or function name.

### Navigation

- index
- modules |
- modules |
- KDVS 0.0.1-alpha documentation »

© Copyright 2010-2012, Grzegorz Zycinski, Salvatore Masecchia, Annalisa Barla.
Created using Sphinx 1.1.2.
