# Supplementary material for: Knowledge Driven Variable Selection (KDVS) – a new approach to enrichment analysis of gene signatures obtained from high–throughput data
Source: Source Code Biol Med. 2013 Jan 9;8:2. doi: 10.1186/1751-0473-8-2 (PMC3605163; doi:10.1186/1751-0473-8-2)
Supplement: Additional file 1 — Source code of KDVS. Format: ZIP. It contains the Python source code, the documentation, and the internal data files. [file 1751-0473-8-2-S1.zip › KDVS/doc/_build/html/index.html]

Knowledge Driven Variable Selection documentation — KDVS 0.0.1-alpha documentation


### Navigation

- index
- modules |
- modules |
- next |
- KDVS 0.0.1-alpha documentation »

# Knowledge Driven Variable Selection documentation¶

|  |  |
| --- | --- |
| Release: | 0.0.1-alpha |
| Date: | September 25, 2012 |

Knowledge Driven Variable Selection (KDVS) provides experimental integration of
biological high-throughput data and prior biological knowledge, performed
with the help of statistical learning algorithms developed by
SlipGURU group at
Dipartimento di Informatica, Bioingegneria, Robotica ed Ingegneria dei Sistemi
(DIBRIS), Università degli Studi di Genova, Italy.

KDVS is licensed under GNU General Public License (GPL) version 3.

KDVS requires Python v2.6+, and the following packages:
:   - numpy – tested with v1.5.1
    - matplotlib – tested with v1.0.1
    - l1l2py – tested with v1.0.5

For (optional) integration with R, it needs:
:   - R – tested with v2.11.1
    - rpy2 – tested with v2.2.3

Contents:

- Overview
  - Idea
  - Input Data
- Methodology
  - Overview
  - Experiment methodology
  - Post-processing methodology
- Applications
  - experiment
  - postprocess
  - pzp\_dump
- Additional topics
  - KDVS Execution Model
  - Parsing metadata
  - PZP
  - PPlus
- KDVS API
  - kdvs.core.GO.GEDM
  - kdvs.core.GO.GOTermTree
  - kdvs.core.GO.GOTermTreeManip
  - kdvs.core.GO.HGNC
  - kdvs.core.GO.annotation
  - kdvs.core.GO.subm
  - kdvs.core.config
  - kdvs.core.db
  - kdvs.core.error
  - kdvs.core.execenv
  - kdvs.core.metadata
  - kdvs.core.provider
  - kdvs.core.rint
  - kdvs.core.util

- *Index*
- *Module Index*
- *Search Page*

### Quick search


Enter search terms or a module, class or function name.

### Navigation

- index
- modules |
- modules |
- next |
- KDVS 0.0.1-alpha documentation »

© Copyright 2010-2012, Grzegorz Zycinski, Salvatore Masecchia, Annalisa Barla.
Created using Sphinx 1.1.2.
