# Supplementary material for: Knowledge Driven Variable Selection (KDVS) – a new approach to enrichment analysis of gene signatures obtained from high–throughput data
Source: Source Code Biol Med. 2013 Jan 9;8:2. doi: 10.1186/1751-0473-8-2 (PMC3605163; doi:10.1186/1751-0473-8-2)
Supplement: Additional file 1 — Source code of KDVS. Format: ZIP. It contains the Python source code, the documentation, and the internal data files. [file 1751-0473-8-2-S1.zip › KDVS/doc/_build/html/np-modindex.html]

Python Module Index — KDVS 0.0.1-alpha documentation


### Navigation

- index
- modules |
- modules |
- KDVS 0.0.1-alpha documentation »

# Python Module Index

**c**

|  |  |  |
| --- | --- | --- |
|  |  |  |
|  | **c** |  |
|  | kdvs.core |  |
|  | kdvs.core.config |  |
|  | kdvs.core.db |  |
|  | kdvs.core.error |  |
|  | kdvs.core.execenv |  |
|  | kdvs.core.execenv.execenv |  |
|  | kdvs.core.execenv.pplus\_env |  |
|  | kdvs.core.GO.annotation |  |
|  | kdvs.core.GO.GEDM |  |
|  | kdvs.core.GO.GOTermTree |  |
|  | kdvs.core.GO.GOTermTreeManip |  |
|  | kdvs.core.GO.HGNC |  |
|  | kdvs.core.GO.subm |  |
|  | kdvs.core.metadata |  |
|  | kdvs.core.provider |  |
|  | kdvs.core.rint |  |
|  | kdvs.core.util |  |

### Quick search


Enter search terms or a module, class or function name.

### Navigation

- index
- modules |
- modules |
- KDVS 0.0.1-alpha documentation »

© Copyright 2010-2012, Grzegorz Zycinski, Salvatore Masecchia, Annalisa Barla.
Created using Sphinx 1.1.2.
