# Supplementary material for: Knowledge Driven Variable Selection (KDVS) – a new approach to enrichment analysis of gene signatures obtained from high–throughput data
Source: Source Code Biol Med. 2013 Jan 9;8:2. doi: 10.1186/1751-0473-8-2 (PMC3605163; doi:10.1186/1751-0473-8-2)
Supplement: Additional file 1 — Source code of KDVS. Format: ZIP. It contains the Python source code, the documentation, and the internal data files. [file 1751-0473-8-2-S1.zip › KDVS/doc/_build/html/search.html]

Search — KDVS 0.0.1-alpha documentation


### Navigation

- index
- modules |
- modules |
- KDVS 0.0.1-alpha documentation »

# Search

Please activate JavaScript to enable the search
functionality.

From here you can search these documents. Enter your search
words into the box below and click "search". Note that the search
function will automatically search for all of the words. Pages
containing fewer words won't appear in the result list.

### Navigation

- index
- modules |
- modules |
- KDVS 0.0.1-alpha documentation »

© Copyright 2010-2012, Grzegorz Zycinski, Salvatore Masecchia, Annalisa Barla.
Created using Sphinx 1.1.2.
