# Supplementary material for: Knowledge Driven Variable Selection (KDVS) – a new approach to enrichment analysis of gene signatures obtained from high–throughput data
Source: Source Code Biol Med. 2013 Jan 9;8:2. doi: 10.1186/1751-0473-8-2 (PMC3605163; doi:10.1186/1751-0473-8-2)
Supplement: Additional file 1 — Source code of KDVS. Format: ZIP. It contains the Python source code, the documentation, and the internal data files. [file 1751-0473-8-2-S1.zip › KDVS/doc/slipGURUTheme/layout.html]

{% extends "basic/layout.html" %}
{% block sidebarsearch %}
{{ super() }}{% endblock %}
{% block extrahead %}


{% endblock %}
{% block sidebarrel %} {% endblock %}
{% block sidebartoc %}
{% if prev %}
{{ super() }}
{% else %}
{% endif %}
{% endblock %}
