# Supplementary figures and images for: Knowledge Driven Variable Selection (KDVS) – a new approach to enrichment analysis of gene signatures obtained from high–throughput data
Source: Source Code Biol Med. 2013 Jan 9;8:2. doi: 10.1186/1751-0473-8-2 (PMC3605163; doi:10.1186/1751-0473-8-2)

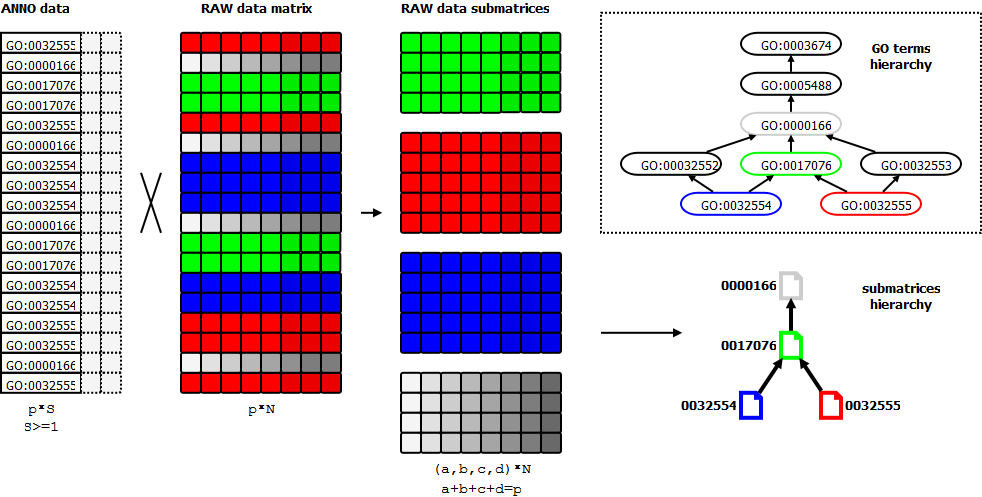

Supplement: Additional file 1 — Source code of KDVS. Format: ZIP. It contains the Python source code, the documentation, and the internal data files. [file 1751-0473-8-2-S1.zip › KDVS/doc/_build/html/_images/gdr1.png]

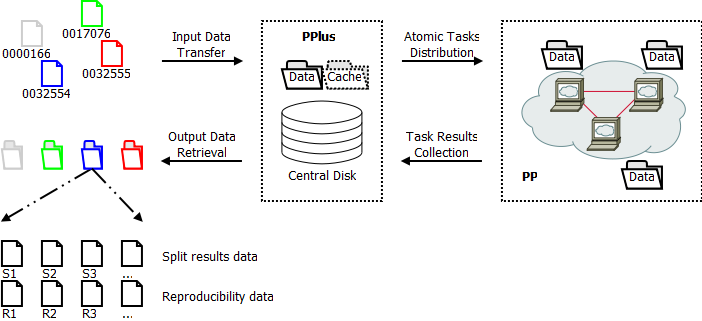

Supplement: Additional file 1 — Source code of KDVS. Format: ZIP. It contains the Python source code, the documentation, and the internal data files. [file 1751-0473-8-2-S1.zip › KDVS/doc/_build/html/_images/gdr2.png]

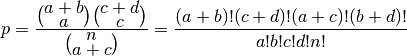

Supplement: Additional file 1 — Source code of KDVS. Format: ZIP. It contains the Python source code, the documentation, and the internal data files. [file 1751-0473-8-2-S1.zip › KDVS/doc/_build/html/_images/math/03459362a61ab22ce9637093ca726af3dcb7da71.png]

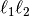

Supplement: Additional file 1 — Source code of KDVS. Format: ZIP. It contains the Python source code, the documentation, and the internal data files. [file 1751-0473-8-2-S1.zip › KDVS/doc/_build/html/_images/math/5123279aac3b29d909bb99bc19d273bb119f8c99.png]

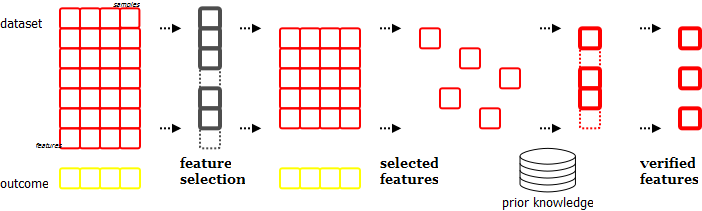

Supplement: Additional file 1 — Source code of KDVS. Format: ZIP. It contains the Python source code, the documentation, and the internal data files. [file 1751-0473-8-2-S1.zip › KDVS/doc/_build/html/_images/meth-1.png]

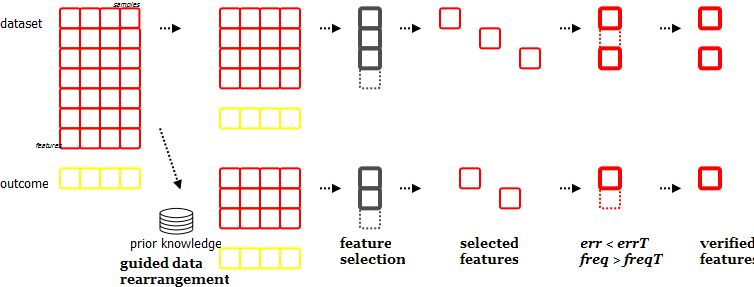

Supplement: Additional file 1 — Source code of KDVS. Format: ZIP. It contains the Python source code, the documentation, and the internal data files. [file 1751-0473-8-2-S1.zip › KDVS/doc/_build/html/_images/meth-2.png]

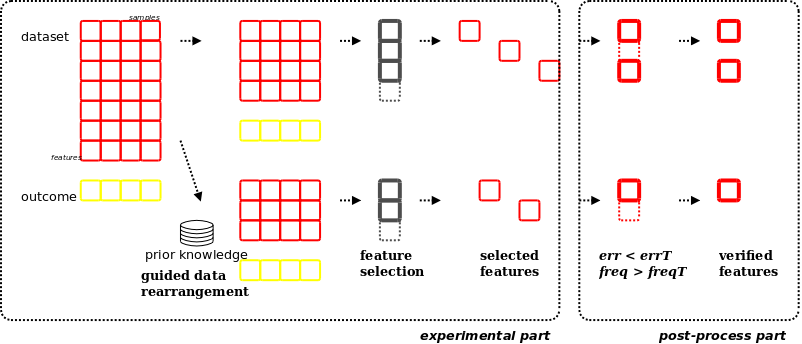

Supplement: Additional file 1 — Source code of KDVS. Format: ZIP. It contains the Python source code, the documentation, and the internal data files. [file 1751-0473-8-2-S1.zip › KDVS/doc/_build/html/_images/meth-3.png]

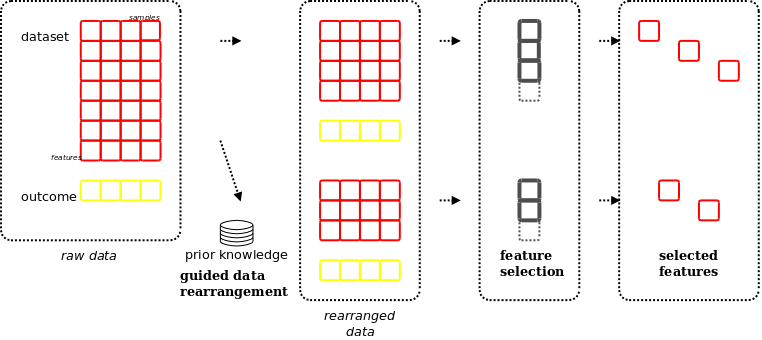

Supplement: Additional file 1 — Source code of KDVS. Format: ZIP. It contains the Python source code, the documentation, and the internal data files. [file 1751-0473-8-2-S1.zip › KDVS/doc/_build/html/_images/meth-exp-1.png]

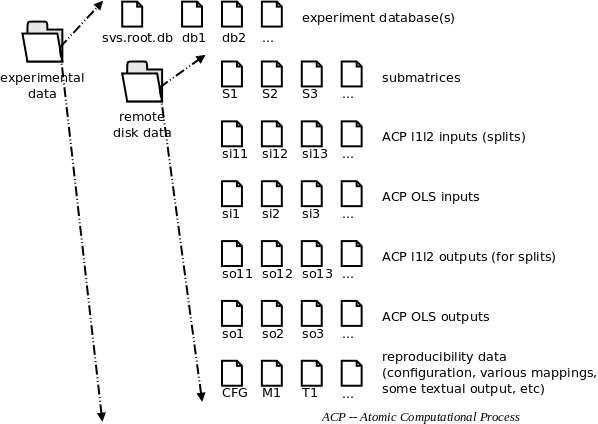

Supplement: Additional file 1 — Source code of KDVS. Format: ZIP. It contains the Python source code, the documentation, and the internal data files. [file 1751-0473-8-2-S1.zip › KDVS/doc/_build/html/_images/post1.png]

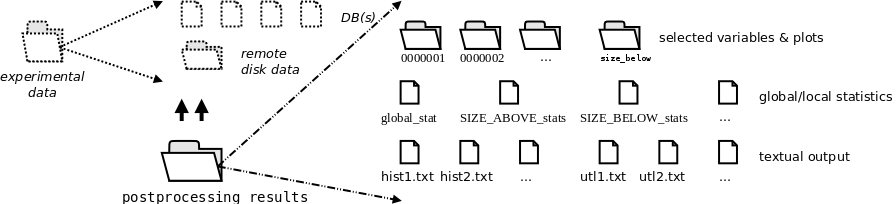

Supplement: Additional file 1 — Source code of KDVS. Format: ZIP. It contains the Python source code, the documentation, and the internal data files. [file 1751-0473-8-2-S1.zip › KDVS/doc/_build/html/_images/post2.png]

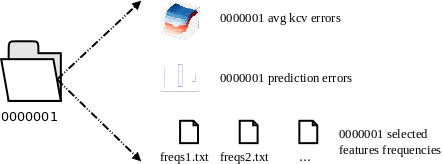

Supplement: Additional file 1 — Source code of KDVS. Format: ZIP. It contains the Python source code, the documentation, and the internal data files. [file 1751-0473-8-2-S1.zip › KDVS/doc/_build/html/_images/post3.png]

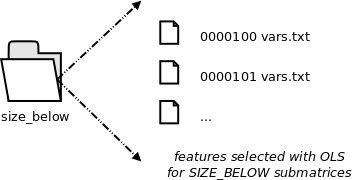

Supplement: Additional file 1 — Source code of KDVS. Format: ZIP. It contains the Python source code, the documentation, and the internal data files. [file 1751-0473-8-2-S1.zip › KDVS/doc/_build/html/_images/post4.png]

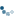

Supplement: Additional file 1 — Source code of KDVS. Format: ZIP. It contains the Python source code, the documentation, and the internal data files. [file 1751-0473-8-2-S1.zip › KDVS/doc/_build/html/_static/ajax-loader.gif]

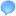

Supplement: Additional file 1 — Source code of KDVS. Format: ZIP. It contains the Python source code, the documentation, and the internal data files. [file 1751-0473-8-2-S1.zip › KDVS/doc/_build/html/_static/comment-bright.png]

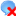

Supplement: Additional file 1 — Source code of KDVS. Format: ZIP. It contains the Python source code, the documentation, and the internal data files. [file 1751-0473-8-2-S1.zip › KDVS/doc/_build/html/_static/comment-close.png]

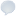

Supplement: Additional file 1 — Source code of KDVS. Format: ZIP. It contains the Python source code, the documentation, and the internal data files. [file 1751-0473-8-2-S1.zip › KDVS/doc/_build/html/_static/comment.png]

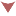

Supplement: Additional file 1 — Source code of KDVS. Format: ZIP. It contains the Python source code, the documentation, and the internal data files. [file 1751-0473-8-2-S1.zip › KDVS/doc/_build/html/_static/down-pressed.png]

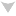

Supplement: Additional file 1 — Source code of KDVS. Format: ZIP. It contains the Python source code, the documentation, and the internal data files. [file 1751-0473-8-2-S1.zip › KDVS/doc/_build/html/_static/down.png]

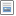

Supplement: Additional file 1 — Source code of KDVS. Format: ZIP. It contains the Python source code, the documentation, and the internal data files. [file 1751-0473-8-2-S1.zip › KDVS/doc/_build/html/_static/file.png]

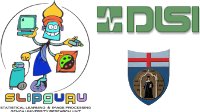

Supplement: Additional file 1 — Source code of KDVS. Format: ZIP. It contains the Python source code, the documentation, and the internal data files. [file 1751-0473-8-2-S1.zip › KDVS/doc/_build/html/_static/logos.png]

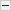

Supplement: Additional file 1 — Source code of KDVS. Format: ZIP. It contains the Python source code, the documentation, and the internal data files. [file 1751-0473-8-2-S1.zip › KDVS/doc/_build/html/_static/minus.png]

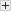

Supplement: Additional file 1 — Source code of KDVS. Format: ZIP. It contains the Python source code, the documentation, and the internal data files. [file 1751-0473-8-2-S1.zip › KDVS/doc/_build/html/_static/plus.png]

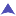

Supplement: Additional file 1 — Source code of KDVS. Format: ZIP. It contains the Python source code, the documentation, and the internal data files. [file 1751-0473-8-2-S1.zip › KDVS/doc/_build/html/_static/up-pressed.png]

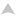

Supplement: Additional file 1 — Source code of KDVS. Format: ZIP. It contains the Python source code, the documentation, and the internal data files. [file 1751-0473-8-2-S1.zip › KDVS/doc/_build/html/_static/up.png]
