# Supplementary material for: Knowledge Driven Variable Selection (KDVS) – a new approach to enrichment analysis of gene signatures obtained from high–throughput data
Source: Source Code Biol Med. 2013 Jan 9;8:2. doi: 10.1186/1751-0473-8-2 (PMC3605163; doi:10.1186/1751-0473-8-2)
Supplement: Additional file 6 — Detailed description of the construction of benchmark data. [file 1751-0473-8-2-S6.pdf]

# Supplementary Material

## Details of construction of benchmark lists for Prostate Cancer and Parkinson's Disease

We have used two benchmark lists to validate the results: gene list and GO term list. In case of both diseases, the benchmark lists were built with identical workflow, depicted on Figure S1. Specific differences for particular diseases are discussed separately.

We compiled the intersection of three individual lists, retrieved from the KEGG DISEASE, KEGG PATHWAY and Gene Prospector databases, queried for related disease. We obtained Gene Ontology Annotations (GOA), compiled for Homo Sapiens, released 25-01-2012. For each gene from intersection, we retrieved related GOA annotations along with their evidences. The *benchmark gene list* was obtained by filtering intersection genes according to evidence strength, explained below.

The *benchmark GO term list* was obtained as follows. For each intersection gene, we obtained associated GO terms, together with their evidences and annotation dates. Then, we filtered GO terms, first according to annotation date, retaining the newer annotations, and secondly according to evidence strength.

Some of the evidences can be more reliable than others. Based on this consideration, we arbitrarily defined the trustability of the evidences, as follows. The evidences recognized as more trustable include all those belonging to the Experimental Evidence Codes (i.e. EXP, IDA, IPI, IMP, IGI, IEP), the Traceable Author Statement (i.e. TAS), and the Inferred by Curator (i.e. IC). We kept the genes and GO terms associated with these more trustable evidences.

For Prostate Cancer, the first individual list consists of genes associated with KEGG DISEASE entry *H00024*, and the second individual list consists of genes associated with KEGG PATHWAY entry *hsa05215*. In both cases, genes come from KEGG GENES database, and are associated via "linkdb". The third individual list consists of the result produced by Gene Prospector for the query "prostate cancer".

For Parkinson's Disease, the first individual list consists of genes associated with KEGG DISEASE entry *H00057*, and the second individual list consists of genes associated with KEGG PATHWAY entry *hsa05012*. In both cases, genes come from KEGG GENES database, and are associated via "linkdb". The third individual list consists of the result produced by Gene Prospector for the query "parkinson's disease".

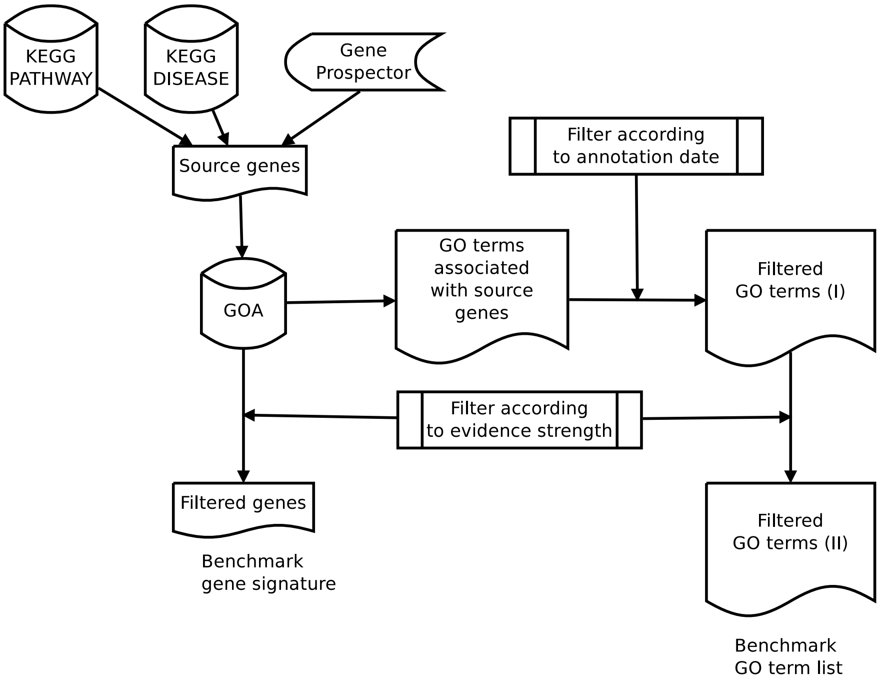


Figure S1: The workflow for obtaining benchmark lists.
